# Multigenerational exposure to trace concentrations of DDT residues in Wistar rats: Effects on biometric development and biochemical parameters

**DOI:** 10.1016/j.toxrep.2025.102012

**Published:** 2025-03-26

**Authors:** Fernanda Coleraus, Camilla de Marchi Sanches Azevedo, Jaine Luana Pavlak, Carla Brugin Marek, Ana Tereza Bittencourt Guimarães

**Affiliations:** a Laboratory of Cellular Toxicology, Center for Medical and Pharmaceutical Sciences, State University of Western Parana (Unioeste), Cascavel, Parana 85819110, Brazil; b Center for Toxicological Information and Assistence (CIATox), University Hospital of Western Parana (HUOP/Unioeste), Cascavel, Parana 85806470, Brazil; c Laboratory of Biological Research, Center for Biological and Health Sciences, Western Paraná State University (Unioeste), Cascavel, Parana 85819-110, Brazil

**Keywords:** Pesticides, Organochlorine, DDD, DDE

## Abstract

The Organochlorine Dichlorodiphenyltrichloroethane (DDT) and its residues, Dichlorodiphenyldichloroethane (DDD) and Dichlorodiphenyldichloroethylene (DDE), are Persistent Organic Pollutants (POPs) that bioaccumulate, persist in the environment, and magnify through the food chain. Chronic exposure is linked to oxidative stress and mitochondrial dysfunction, emphasizing the need to study its multigenerational impacts on health and development. This study investigated the effects of multigenerational exposure to DDT residues in Wistar rats. Pregnant females were provided water containing trace concentrations of p,p’-DDD (0.015 µM) and p,p’-DDE (0.006 µM) from the first day of gestation (PD0) until the end of the life cycle of two generations (F1 and F2). Biometric and biochemical evaluations were conducted at PND35 and PND105, including weight, naso-anal length, and abdominal circumference. Hepatic, renal, and adipose tissues were analyzed macro- and microscopically, along with biochemical analyses. Statistical analyses included ANOVA and generalized linear models. The hypothetical model confirmed that no significant variations occurred between generations, indicating that effects were driven by group, age, and sex differences. The analysis revealed that DDD/DDE synergism and female sex significantly influenced hepatic, renal, cerebral, and white adipose tissues. DDD/DDE exposure increased hepatic enzyme activity, reduced cerebral cholinesterase and renal antioxidants, and altered adipocyte mass. Age also influenced enzymatic activity and development, with notable differences between PND35 and PND105 in tissues and biometric indices. In conclusion, DDD/DDE exposure, particularly in females, significantly impacted hepatic, renal, cerebral, and adipose tissues. The results highlight that observed effects depend on group, age, and sex, emphasizing the risks associated with environmental contamination.

## 1 Introduction

The organochlorine dichlorodiphenyltrichloroethane (DDT) and its residues, dichlorodiphenyldichloroethane (DDD) and dichlorodiphenyldichloroethylene (DDE), are Persistent Organic Pollutants (POPs) that bioaccumulate in organisms, persist in the environment, and magnify through the food chain [21], [30], [43], [76]. The commercial formulation of DDT primarily consists of the p,p’-DDT isomer (70 %), along with its byproducts, p,p’-DDD and p,p’-DDE [74].

Fernandes et al. [21] report the presence of DDT and its residues, along with 55 other pesticides, in soils from residential areas in Brazil. Corroborating these findings, Wolfart et al. [83] also detect trace concentrations of DDT and its residues in Brazilian soil samples. Furthermore, another study indicates that environmental contamination has resulted in DDT residue concentrations exceeding the limits established by WHO drinking water guidelines in several African countries [43].

The persistence of DDT and its residues in soil [21], [83], water [43], and human reservoirs [44] is attributed to their high lipophilicity, slow metabolism, and predominant storage as DDE in lipid-rich tissues [1], [25], [55], [5].

Research shows that chronic exposure to DDT and its residues, especially DDE, induces oxidative stress, mainly in mitochondria, as a key mechanism of toxicity linked to diseases and cancer [27], [3], [49]. Elevated DDE levels increase oxidative stress, causing protein oxidation, DNA damage, and lipid peroxidation [9]. DDT and DDE also disrupt mitochondrial activity by reducing ATP production and blocking proton transport at complex V (ATPase), impairing ADP phosphorylation [18]. This disruption leads to excess electrons in the electron transport chain, forming reactive oxygen species (ROS).

Studies also show that DDD and DDE stimulate cytochrome P450 in a dose- and sex-dependent manner, leading to excessive production of reactive oxygen species (ROS) [27], [71]. Cytochrome P450 enzymes catalyze redox reactions that produce highly reactive metabolites, such as epoxides, DDMU-epoxide, and DDE-epoxide, from DDD and DDE. These metabolites are highly reactive and strongly bind to nucleophilic sites in proteins and DNA [48].

These changes have been linked to cytotoxic [58], [59], nephrotoxic [24], [46], hepatotoxic (Migliaccio et al., 2019; [52], [67]), and neurotoxic effects [11], [19], [31], [66], as well as increased body fat and changes in biometrical development [36], [60].

However, despite recent studies highlighting the alarming presence of DDT and its residues in soil and water samples across various regions worldwide [21], [43], [44], [83]. When considering multiple contaminants and multigenerational exposure to residual pollutants over time, establishing clear cause-and-effect relationships for human health becomes complex and challenging. To this end, the study was designed to evaluate the development of biometric and biochemical alterations resulting from multigenerational exposure to trace concentrations of DDT residues, both individually and synergistically. It also aimed to examine the potential consequences for the descendants of the first and second generations at the beginning of puberty and during adulthood of the animals.

## 2 Methods and materials

To determine the effects of DDT residues on the offspring of rats exposed during the pre- and postnatal periods, tap water containing the formulations was provided ad libitum from the first day of gestation (PD0) until the end of the life cycle of two generations.

The experimental protocol followed the guidelines established by the National Council for the Control of Animal Experimentation (CONCEA) and was approved by the Animal Use Ethics Committee (CEUA) of Unioeste, Brazil (protocol no. 21–20; Annex 1 and 2).

### 2.1 Water formulated with trace concentrations of DDT residues

To simulate a population exposed to DDT residues, trace concentrations were determined based on studies examining DDT residues in soil samples [21], [83]. Standards of p,p’-DDD (1,1-Dichloro-2,2-bis(4-chlorophenyl)ethane) and p,p’-DDE (1,1-Dichloro-2,2-bis(4-chlorophenyl)ethylene) were obtained from Sigma Chemical Co. (St. Louis, MO, USA), dissolved in ethanol (99.5° GL, Sigma Chemical Co.), and stored at 4°C in light-protected conditions. These standards were added to the drinking water provided ad libitum to the animals throughout the experiment, resulting in final concentrations of 0.005 mg·L⁻¹ p,p’-DDD (0.015 µM) and 0.002 mg·L⁻¹ p,p’-DDE (0.006 µM). To standardize conditions, the water supplied to both control and treated groups was adjusted to contain 0.01 % ethanol as the vehicle [60].

### 2.2 Experimental design

**Fig. 1:**
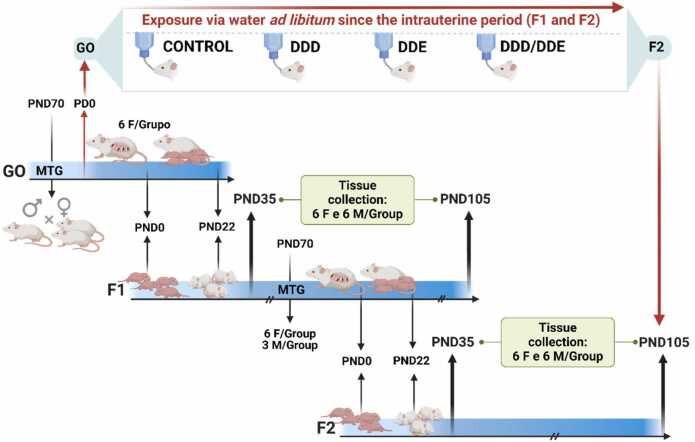
Experimental design. g0 – Parental generation; F1 – First generation; F2 – Second generation; PND – Postnatal days; PD0 – Pregnancy day 0; F – Females; M – Males; ACS – Mating; F – Females; M – Males. Created in BioRender. Bittencourt guimarães, A. (2025) https://BioRender.com/b09u490.

The experiment followed a completely randomized design with a factorial scheme, including the factors DDD exposure (non-exposed and exposed), DDE exposure (non-exposed and exposed), age (PND35 and PND105), and generation (F1 and F2). It began with 36 Wistar albino rats (24 females and 12 males), aged 56 postnatal days (PND), non-consanguineous, which were mated and designated as the parental generation. The study then continued with the offspring from two descendant generations (F1 and F2) of the parental generation (Fig. 1).

The animals were housed in polypropylene cages under controlled conditions (22°C ± 2°C, 12-hour light/dark cycle, and air exhaustion), fed a standard rodent diet, and given potable water *ad libitum*. The experiment was conducted at the Sectoral Animal Facility of the State University of Western Parana (Unioeste, Cascavel, PR, BR). The protocol complied with guidelines from the National Council for the Control of Animal Experimentation (CONCEA) and was approved by UNIOESTE’s Ethics Committee on Animal Use (CEUA; protocol no. 21–20).

#### 2.2.1 Parental generation

After a 10–14 day acclimation period at the Sectoral Animal Facility, 24 females and 12 males aged PND70 were housed for mating at a ratio of 2 females to 1 male. The animals were kept together for 5 days (the duration of the estrous cycle). Pregnancy confirmation (day PD0) was determined by detecting spermatozoa in vaginal smears from the females. Once pregnancy was confirmed, the males were euthanized using an intraperitoneal overdose of anesthetics (Ketamine 300 mg·kg⁻¹ and Xylazine 45 mg·kg⁻¹).

On PD0, pregnant females were randomly divided into four groups (n = 6 per group) and were provided tap water ad libitum as follows: **CTL (Control) group:** Provided tap water; **DDD group:** Provided tap water containing 0.015 µM of p,p’-DDD; **DDE group:** Provided tap water containing 0.006 µM of p,p’-DDE; **DDD/DDE group:** Provided tap water containing 0.015 µM of p,p’-DDD and 0.0056 µM of p,p’-DDE.

At the end of the lactation period, vaginal smears were prepared for the parental generation females as described by Marcondes et al. [45] to confirm the estrous phase of the cycle. Once the estrous phase was confirmed, the females were euthanized using an intraperitoneal overdose of Ketamine (300 mg·kg⁻¹) and Xylazine (45 mg·kg⁻¹).

The offspring of the parental generation were designated as the F1 generation, and their descendants were designated as the F2 generation.

#### 2.2.2 Descendant generations (F1 and F2)

After weaning on PND22, the F1 generation offspring were sexed, and the number of animals was standardized to 18 females per group (3 females per litter) and 15 males per group (2–3 males per litter). This distribution was necessary to conduct the F1 generation evaluation, which included three stages: a mating stage to produce the F2 generation and two evaluation phases (PND35 and PND105).

The F2 generation was obtained using the same method as the parental generation, with the number of offspring standardized to 24 per group (2 females and 2 males per litter), resulting in 12 females and 12 males in each group.

To examine the cumulative and multigenerational effects of DDT residue exposure, the F1 and F2 offspring followed the same treatment protocol as the parental generation. They were provided *ad libitum* water formulations containing DDT residues according to their respective groups (CTL, DDD, DDE, DDD/DDE) throughout their life cycle. Both generations (F1 and F2) were evaluated during two distinct phases over a total period of 105 days.

### 2.3 Tissue collection and morphological analysis

Males (n = 6 per group) and females (n = 6 per group) from the F1 and F2 generations were sampled at PND35 (onset of puberty) and PND105 (adult stage). The animals were anesthetized with an intraperitoneal injection of Ketamine (100 mg·kg⁻¹) and Xylazine (15 mg·kg⁻¹), followed by exsanguination via cardiac puncture.

The brain, liver, kidneys, and fractions of visceral adipose tissue (VAT) and perigonadal adipose tissue (PAT) from white adipose tissue (WAT) were collected, weighed, and examined macroscopically. The tissues were then placed in microtubes, flash-frozen in liquid nitrogen, and stored at −80°C for subsequent biochemical analyses.

### 2.4 General characterization

During tissue collection, any observed macroscopic morphological alterations were documented, and a portion of the affected tissue was preserved in a 10 % formalin solution. These samples were sent for histological analysis to a supporting histopathology laboratory (LabPat, Unioeste, Cascavel, PR, Brazil).

For biometric analysis, weight, naso-anal length (NAL), and abdominal circumference (AC) were measured immediately after weaning (PND22) and re-evaluated weekly until euthanasia (PND35 or PND105). Weekly records of food and water intake were also maintained. Using the collected biometric data, the following indices were calculated:

*Eq. 1. Body Weight Gain*: $\mathrm{BWG}\left(g\right)=\mathrm{Final Weigth}-\mathrm{Initial Weight}$*.*

*Eq. 2. Abdominal circumference gain:* ΔAC(cm)=AC final−AC initial *.*

*Eq.3. Body length:* ΔNAL(cm)=NAL final−NAL initial *.*

*Eq.4. Specific Weight Gain Rate:* WGR=∆Weight/Initial Weight

Eq.5. Final Lee Index: $\mathrm{LI}=\;\frac{\sqrt[{3}]{\mathrm{Final Weight}}\;(g)}{\mathrm{NAL final}(\mathrm{cm})}*1000$.

*Eq.6. Calculation of Somatic Indices:* $\mathrm{SI}(\%)=\;\frac{\mathrm{Tissue Weight}(g)}{\mathrm{Final Body Weight}(g)}*100$ *.*

*Eq.7. Total Food Consumption:* $\mathrm{FC Total}(g)=\;\sum {\left(\mathrm{Weekly Feed Intake}\right)}_{\mathrm{per group}}$

*Eq.8. Total Water Consumption:* $\mathrm{WC Total}(\mathrm{mL})=\;\sum {\left(\mathrm{Weekly Water Intake}\right)}_{\mathrm{per group}}$

Eq.9. Weight Gain-to-Caloric Intake Ratio: $\mathrm{WGTIR}\left(g/\mathrm{kcal}\right)=\frac{\Delta \mathrm{Weight}}{\left(\mathrm{FC Total}*3,8\mathrm{kcal}/g\right)}$

### 2.5 Biochemical analysis

The tissues were homogenized in Tris-HCl buffer (50 mM, pH 7.4) at a volume equal to five times the tissue weight. Following homogenization, cellular debris was removed through differential centrifugation (10 min at 10,260 g, 4°C). Aliquots of the resulting supernatants (homogenates) were then stored in microtubes.

Total protein concentrations in the tissues were measured using the Bradford [6], with bovine serum albumin (Sigma-Aldrich, MO, USA) as the standard. Based on the protein concentrations required for each specific analysis, the samples were normalized using Tris-HCl buffer (50 mM, pH 7.4).

Biochemical analyses included measuring the activities of the following enzymes in hepatic and renal tissues: Superoxide Dismutase (SOD, [12]), Catalase (CAT, [2]), Glutathione Peroxidase (GPx, Flohé and Günzler, 1984), Glutathione-S-Transferase (GST, [33]), and Glutathione Reductase (GR, [72]). The hepatic enzyme Glutamic Pyruvic Transaminase (GPT) was measured using a commercial kit (Gold Analisa, MG, Brazil).

Cholinesterase activity was assessed in hepatic tissue (BuChE) and brain tissue (AChE) following the method of Ellman [17]. Oxidative analysis of membrane lipids (LPO – Lipoperoxidation) in VAT, PAT, hepatic and renal tissues was conducted by measuring malondialdehyde (MDA) levels, a lipid degradation product, using the thiobarbituric acid-reactive substances (TBARs) method described by Draper and Hadley [16].

### 2.6 Statistical analysis

All analyses were conducted using R software [62]. Variables were first evaluated for normality using the Shapiro-Wilk test and for homogeneity of variances using Levene's test. Variables with a normal distribution and homogeneous variances were analyzed using a three-way ANOVA, considering the fixed factors: Generation (F1 and F2), Age (PND35 and PND105), and Group (CTL, DDD, DDE, and DDD/DDE). When statistical significance was detected (p < 0.05), post hoc multiple comparisons were performed using the Tukey HSD test. For variables that did not meet the assumptions of normality or homogeneity, generalized linear models (GLM) with a Gamma distribution were applied, using generation, age, and group as predictor variables.

Subsequently, the variables, which exhibited linear relationships, were standardized as z-scores and analyzed using the Partitioning technique. This method involves pre-modeling by combining multiple variables, using factor loadings from Principal Component Analyses (PCA) based on relationships within biological systems. Partitioning offers significant advantages, retaining original variables while incorporating the characteristics of their respective domains [70], [41]. Table 1 outlines the partitioning and the items included in each partition.

**Table 1:** Variables analyze using the partitioning technique.

**Parcel**	**Variables**
Cerebral Response	AChE; SI.
Hepatic Response	LPO; SOD; CAT; GPx; GR; GST; SI; TGP; BuChE.
Renal Response	LPO; SOD; CAT; GPX; GST; GR; SI.
White Adipose Tissue Response	LPO-VAT; LPO-PAT; SI-VAT; SI-PAT.
Body Development	∆AC; ∆NAL; WGR; LI; WGTIR.

**Fig. 2:**
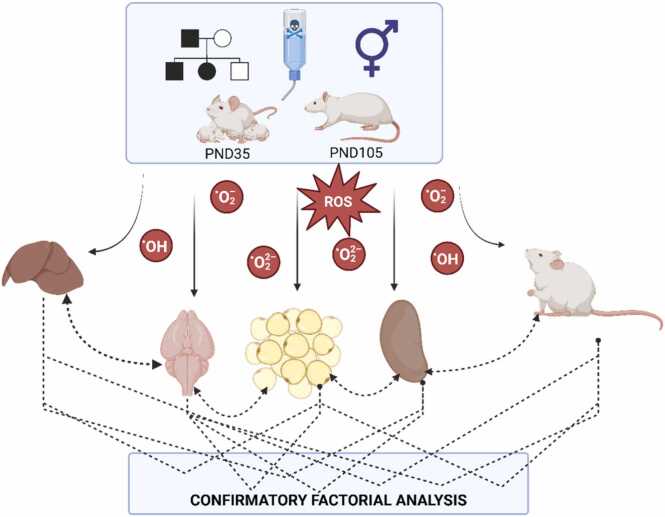
Hypothetical model of tissue response influenced by group, sex, age, and generation factors. The factors considered statistically significant standardized weights supporting the proposed model were categorized as Group (CTL – 0, DDD – 1, DDE – 2, DDD/DDE – 3), Sex (M – 1, F – 2), Age (PND35 – 1, PND105 – 2), and Generation (F1 – 1, F2 – 2). Created in BioRender. Bittencourt guimarães, A. (2025) https://BioRender.com/b09u490.

The first two principal components of the parcels were incorporated into the hypothetical model (Fig. 2), where Confirmatory Factor Analysis was applied. Statistically significant standardized weights were used to support the hypothesis that the factors Group (CTL: 0, DDD: 1, DDE: 2, DDD/DDE: 3), Sex (M: 1, F: 2), Age (PND35: 1, PND105: 2), and Generation (F1: 1, F2: 2) influence tissue responses under multigenerational contamination conditions. The weights can be used to quantify the variability of the variables that can be explained by the underlying factor. The weights can be used to quantify the variability of the variables that can be explained by the underlying factor. The higher the standardized weight, the greater the influence of the factor. The weights can be interpreted the same way as the correlation coefficients, and they estimate the association between the factors and the variables.

Several fit measures were used to test the models. The chi-square test examines whether a model significantly deviates from a perfect fit to the data. Since this test is highly dependent on sample size, the following model fit indices were used: Comparative Fit Index (CFI), Standardized Root Mean Square Residual (SRMR), and Root Mean Square Error of Approximation (RMSE). A CFI of 1 indicates a perfect fit, and values of 0.9 indicate a good fit. For SRMR, the closer the value is to 0, the better the model fit. For RMSE, values below 0.05 indicate a good fit. Finally, the Akaike Information Criterion (AIC) was used for comparisons between models that included different variables. Models with the lowest AIC are considered the best fit.

## 3 Results

### 3.1 General characterization

**Fig. 3:**
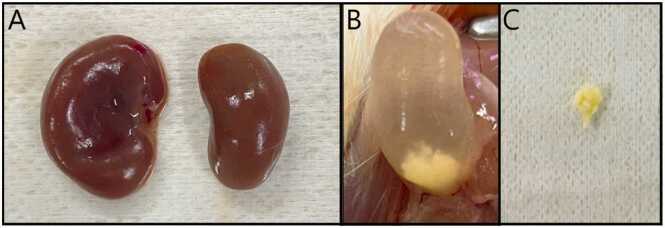
Macroscopic morphological changes in renal tissue and the presence of urinary lithiasis in rats exposed to residues of DDT. (A) The photo shows the right kidney (1.76 ×1.2 cm; 0.842 g) and left kidney (1.48 ×0.89 cm; 0.530 g) of an animal from the DDD group, PND35, F1 generation, male sex; (B) Bladder of an animal from the DDE group, PND105, F1 generation, male sex, presenting a calculus; (C) Calculus extracted from the bladder of an animal from the DDE group, PND105, F1 generation, male sex.

In the tissue analysis, macroscopic morphological changes were observed in the renal and hepatic tissues at both ages and in both generations. Fig. 3A illustrates renal hypertrophy in a male from the F1 generation at PND35 exposed to DDD. At PND105, urinary lithiasis was identified in males from both generations (Fig. 3B and C), with an incidence of 44 % (χ² = 7.45; p = 0.0590) among males exposed to DDE.

**Fig. 4:**
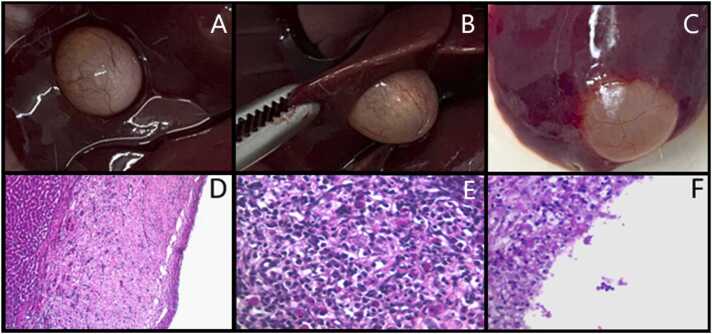
Benign hepatic nodule in rats exposed to residues of DDT at PND105 in F2 generation. Figures A-C show sessile nodules measuring approximately 343 mm³ , with a translucent surface and serous-looking liquid content found in an animal from the DDD/DDE group, PND105, F2 generation, female (A-B) and in an animal from the DDE group, PND105, F2 generation, male (C). Figures D-F show the histological analysis of the hepatic nodule, with a cavity surrounded by dense fibrous connective tissue (D), intense angiogenesis, chronic plasmocytic inflammation with Russell bodies and eosinophils (E), and the presence of foamy macrophages at the lumen cavity border and mucous material (F).

In hepatic tissue analysis, benign hepatic nodules were observed in a female from the DDE group and a male from the DDD/DDE group, both from the F2 generation, at PND105 (Fig. 4). The hepatic nodules shared similar characteristics, described as benign hepatic nodules, sessile, with a translucent surface and containing serous-like fluid. Histological examination revealed dense fibrous connective tissue with intense angiogenesis, chronic plasmacytic inflammation with Russell bodies, eosinophils, foamy macrophages, and mucous material in both tissues.

Evaluating the biometric measures of female rats (Table S1), significant differences were observed in FC_Total_ (F_3_ = 4.1485; p = 0.0081), WC_Total_ (F_3_ = 31.2344; p < 0.0001), and WGTIR (F_3_ = 14.5068; p < 0.0001) among groups exposed to residues of DDT. In the FC_Total_ analysis, the DDE group at PND105, regardless of generation, showed a significant reduction in food intake (p = 0.0155) compared to the Control. While no significant changes in FC_Total_ were observed in the other groups, analysis of WGTIR at PND35 and PND105, regardless of generation, revealed notable differences. In the DDD group at PND35, the coefficient significantly decreased (p = 0.0066), whereas the DDD/DDE association at PND35 caused a significant increase in the coefficient (p < 0.0001) compared to the control.

In the analysis of water consumption among female rats, significant variations were observed among the groups exposed to DDT residues. A significant reduction in WC_Total_ was found in animals exposed to DDE at PND105 in generation F1 (p < 0.0001), while a significant increase in WC_Total_ was observed in the DDD/DDE combination group (p < 0.0001) compared to the control.

Table S2 (Supplementary Material) presents the biometric measures of male rats. Significant changes were observed among the groups, regardless of age and generation, in renal SI (F_3_ = 5.3445; p = 0.0736), WGR (F_3_ = 2.9268; p = 0.0377), WGTIR (F_3_ = 2.7844; p = 0.0450), and WC_Total_ (F_3_ = 3.2743; p = 0.0244). Renal SI significantly decreased in the DDE group (p = 0.0736) compared to the control. In the analysis of WGR and WGTIR, although significant variations occurred between groups, the follow-up statistical test identified differences only among the groups exposed either individually or combined with DDD/DDE, but no statistical differences were found when compared to the control group. Other parameters did not show significant variations among the groups.

In the evaluation of water consumption among male rats, it was observed that all groups of generation F1, exposed to DDT residues, had their WC_Total_ reduced (p < 0.0001) compared to the control. When associating group, generation, and age, it was identified that the significant reduction in WC_Total_ occurred in all F1 groups at PND105, exposed to DDT residues (p < 0.0001), compared to the control, with no such reduction observed in F2.

The evaluation of the animals' water consumption throughout the entire experiment was crucial to obtain the average daily consumption of DDD and DDE by the animals. Thus, evaluating the daily consumption of DDD and DDE relative to the weight of each animal and considering both groups, generations, ages, and sex, the average daily consumption of DDD was 0.803 µg.kg^−1^.day^−1^ (95 % CI: 0.789 – 0.818), and for DDE, it was 0.299 µg.kg^−1^.day^−1^ (95 % CI: 0.294 – 0.304).

### 3.2 Biochemical parameters

In the individual analysis of the biochemical parameters of females (Table S3), when evaluating differences between groups regardless of age and generation, a reduction in renal CAT levels was observed in the DDD group (p = 0.0308) compared to the control. No significant variations were identified in the other variables among the treated groups compared to the control. When analyzing groups within each generation, regardless of age, animals exposed to DDE in the F2 generation showed a significant increase in LPO in PAT (p = 0.0020), as well as increases in SOD (p = 0.0146) and GPx (p = 0.0072) in renal tissue compared to Control group. Considering age, regardless of generation, the DDE group at PND105 continued to exhibit increased LPO in PAT (p = 0.0011) and elevated GPx (p = 0.0037) in renal tissue compared to Control group. No significant variations were observed when evaluating the relationship between group, age, and generation, likely due to the reduced number of animals per group when these three factors were combined.

In males (Table S4), when evaluating the biochemical parameters between treated groups compared to the control, regardless of age and generation, animals exposed to DDD showed a reduction in hepatic LPO levels (p = 0.0046) and an increase in LPO levels in PAT (p = 0.0122) relative to the control. The increase in LPO in PAT remained significant when considering group and age, specifically in animals exposed to DDD at PND35 (p = 0.0177). In the DDE group, regardless of age and generation, a significant reduction in hepatic SOD enzyme activity was observed (p = 0.0241), along with an increase in GPx enzyme activity in renal tissue (p = 0.0050). The remaining variables, when analyzed individually, did not show significant variations.

### 3.3 Integrative analysis

Considering that in an organism, systems work in an integrated manner, it is necessary to evaluate the biological variables through Correlation Analysis using Principal Component Analysis (PCA). This analysis allows for summarizing a large group of variables into a reduced number of latent variables, while still enabling an integrated interpretation of the behavior of a biological system.

**Fig. 5:**
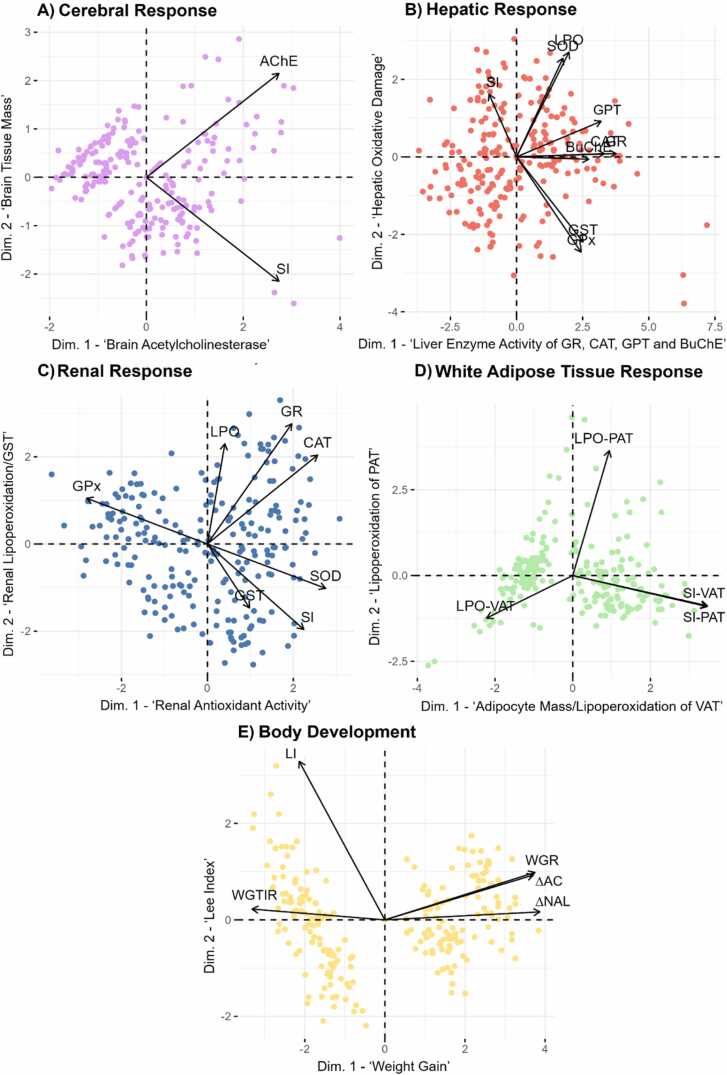
Principal component analysis (PCA) ordination diagrams. A) Cerebral response; B) Hepatic response; C) Renal response; D) White adipose tissue response; E) Body development. Dim.1 – Dimension 1; Dim. 2 – Dimension 2. AChE – Cerebral Cholinesterase; BuChE – Butyrylcholinesterase; CAT – Catalase; FC Total – Total food consumption; GPx – Glutathione Peroxidase; GR – Glutathione Reductase; GST – Glutathione-S-Transferase; GPT – Glutamic Pyruvic Transaminase; LI – Lee index; LPO – Lipoperoxidation; PAT – Perigonadal adipose tissue; SI – Somatic index of each organ; SOD – Superoxide Dismutase; VAT – Visceral adipose tissue; WC Total – Total water consumption; WGTIR – Weight Gain-to-Caloric Intake Ratio; WGR – Specific weight gain rate; ∆AC – Abdominal circumference gain; ∆NAL – Naso-anal length gain.

In the evaluation of the brain tissue, which presents only two variables, AChE and SI, PCA was exclusively required to summarize the variables into factor loadings and showed a positive association between ‘Brain Acetylcholinesterase’ (Dimension 1 – Dim. 1) and ‘Brain Tissue Mass’ (Dimension 2 – Dim. 2; Fig. 5A).

The analysis of hepatic tissue (Fig. 5B) in its first principal component (Dim.1) showed the greatest contribution from the variables GR, CAT, GPT, and BuChE, all being directly related, with the highest values represented by the positive scores of the factor loadings. The latent variable generated in Dim. 1 can be termed as ‘Hepatic Enzyme Activity of GR, CAT, GPT, and BuChE’ (Eigenvalue = 3.30; Variance = 36.70 %). The second principal component (Dim. 2) showed a greater contribution from the variables SI, LPO, SOD, GST, and GPx, with the first three variables being directly related and represented by positive scores, while the last two were inversely related to the first three and represented by negative scores. Thus, Dim. 2 can be termed as ‘Hepatic Oxidative Damage’ (Eigenvalue = 1.47; Variance = 16.34 %).

The analysis of renal tissue (Fig. 5C) showed that in the first principal component (Dim.1), SOD, CAT, SI, and GPx had the greatest contribution, with positive scores indicating an increase in SOD and CAT and a reduction in GPx. The latent variable generated in Dim. 1 can be termed as ‘Renal Antioxidant Activity’ (Eigenvalue = 2.09; Variance = 29.85 %). The second principal component (Dim. 2) showed a greater contribution from the variables GR, LPO, and GST, with the first two being directly related and represented by positive scores, while GST was inversely related to the first two and represented by a negative score. Thus, Dim. 2 can be termed as ‘Renal Lipoperoxidation/GST’ (Eigenvalue = 1.64; Variance = 23.40 %). The variable SI was present and contributed equally to both dimensions 1 and 2.

The analysis of WAT (Fig. 5D) showed that in the first principal component (Dim.1), the greatest contribution came from LPO and SI of the VAT and SI of the PAT, with negative scores indicating a reduction in LPO in VAT and positive scores indicating an increase in the somatic indices of both tissues. The latent variable generated in Dim. 1 can be termed as ‘Adipocyte Mass/Lipoperoxidation of VAT’ (Eigenvalue = 1.83; Variance = 45.68 %). The second principal component (Dim. 2) showed a greater contribution from the LPO variable of PAT, with its increase represented by the positive score, and can be termed as ‘Lipoperoxidation of PAT’ (Eigenvalue = 1.01; Variance = 25.16 %).

Body development (Fig. 5E) analysis revealed that the first principal component (Dim. 1) was primarily influenced by ∆NAL, WGR, and ∆AC (positive scores), and WGTIR (negatively correlated). The latent variable generated in Dim. 1 can be termed as ‘Weight Gain’ (Eigenvalue = 4.64; Variance = 77.32 %). The second principal component (Dim. 2), driven solely by the LI (positive score), represents the 'Lee Index' (Eigenvalue = 0.87; Variance = 14.52 %).

**Fig. 6:**
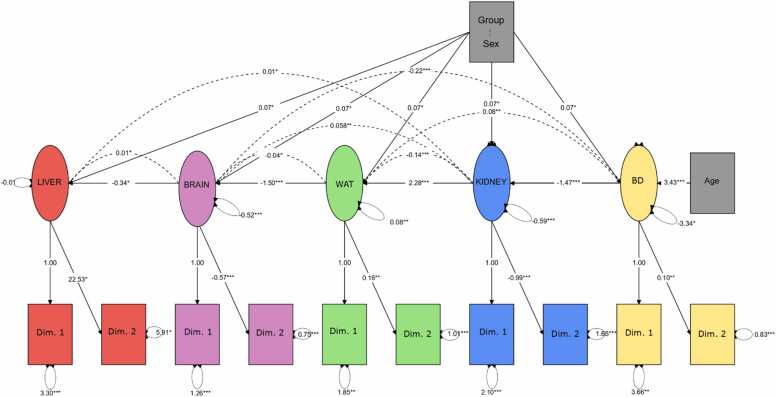
Confirmatory analysis of the hypothetical model of each tissue's response to the influence of the group, sex, age, and generation factors. The statistically significant standardized weights that support the proposal were categorized as Group (CTL – 0, DDD – 1, DDE – 2, DDD/DDE – 3), Sex (M – 1, F – 2), and Age (PND35 – 1, PND105 – 2). WAT: White Adipose Tissue; BD: Body Development. Lines represent cause-and-effect relationships, and dashed lines represent associations between the effects in each tissue. *p < 0.05; * *p < 0.01; * **p < 0.001; * ** *p < 0.0001.

After defining the first two principal components for each tissue plot and including them in the hypothetical model, a confirmatory factorial analysis was performed (Fig. 6). For the analysis, the factors used in the proposal were: Group (CTL – 0, DDD – 1, DDE – 2, DDD/DDE – 3), Sex (M – 1, F – 2), Age (PND35 – 1, PND105 – 2), and Generation (F1 – 1, F2 – 2). The observed responses in the tissues were considered as cause-and-effect relationships or associations, being directly (positive coefficients) or inversely (negative coefficients) proportional. Based on the analysis, it was found that the 'generation' factor was not significant, as the results observed between the generations were equivalent (p > 0.05). The hypothetical model without the ‘generation’ factor showed a good fit, confirming this new proposal (CFI = 0.766; SRMR = 0.164; RMSE = 0.225; AIC = 6144).

It was possible to observe that exposure in the order DDD < DDE < DDD/DDE, especially in females, significantly influenced all tissues. In the hepatic tissue, the synergy of DDT residues, particularly in females at the beginning of puberty (PND35), led to an increase in the ‘Activity of hepatic enzymes GR, CAT, GPT, and BuChE’ (Dim.1, p < 0.0001). In the ‘Hepatic oxidative damage’ dimension, despite observing a trend, no significant relationships were found (Dim. 2; p = 0.079).

In renal tissue, exposure to DDD and DDE, particularly in females at the beginning of puberty (PND35), showed an inverse and significant relationship in ‘Renal antioxidant activity’ (Dim. 1; p < 0.0001). There was also a significant inverse relationship with ‘Renal Lipoperoxidation/GST’ (Dim. 2; p < 0.0001).

In the brain tissue, the synergy between DDD and DDE, in females at the beginning of puberty (PND35), showed an inverse and significant relationship, resulting in a reduction in the activity of ‘Brain Acetylcholinesterase’ (Dim.1, p < 0.0001) and the ‘Brain tissue mass’ (Dim.2, p < 0.0001) in the tissue.

The observed effects in WAT and development were influenced by the DDD/DDE synergy, and the model also showed that exposure in females during adulthood (PND105) significantly influenced tissue responses. In WAT, a direct and significant relationship was observed with ‘Adipocyte mass/Lipoperoxidation in VAT’ (Dim. 1; p = 0.001) and with ‘Lipoperoxidation of PAT’ (Dim. 2; p < 0.0001).

In the analysis of interactions between variables related to the animals' development, the synergy of DDD and DDE in adult females (PND105) showed a direct and significant relationship with 'Weight gain' (Dim. 1; p < 0.0001). Furthermore, there was a direct and significant relationship with the ‘Lee Index’ (Dim. 2; p < 0.0001).

Thus, the results of the hypothetical model showed that the DDD/DDE synergy and the sex factor, in the order male < female, were related to the effects observed in all tissues analyzed. As for the age factor, the order PND105 < PND35 was associated with increased hepatic enzyme activity, reduced cholinesterase and somatic index in brain tissue, reduced antioxidant activity and renal lipoperoxidation, as well as increased renal GST. In WAT and development, the age order PND35 < PND105 was related to increased adipocyte mass, lower lipoperoxidation in VAT, higher lipoperoxidation in PAT, weight gain, and the Lee Index.

## 4 Discussion

Aspects of biotransformation, bioaccumulation, and biomagnification processes contribute to the persistence of DDT residues, particularly DDE, in animals [34], in the human body [8], and in the environment [43], even decades after restrictions were imposed on the product [76]. Thus, threshold doses of daily exposure have been established to ensure the safety of the population in rural and urban areas, which are known to be contaminated with POPs, such as DDT and its residues [10], [4].

Daily exposure limits are generally obtained through estimates based on key concentrations obtained in experiments, thus defining the values possibly safe for human exposure. Until the last publication from the Agency for Toxic Substances and Disease Registry (2022), limits of 0.01 mg.kg^−1^.day^−1^ for the p,p-DDD isomer and 1 mg.kg^−1^.day^−1^ for the p,p-DDE isomer, both administered orally, were defined as the lowest values capable of causing any adverse effects in animal models (Low Observed Adverse Effect Level – LOAEL). However, the study revealed that the ingestion of daily doses 10-fold below (0.0008 mg.kg-1.day-1) the LOAEL for p,p’-DDD and 3,000-fold below (0.0003 mg.kg-1.day-1) the LOAEL for p,p’-DDE was sufficient to cause alterations in the tissues and development issues in animals.

The confirmatory factor analysis of the hypothetical model indicated that the factor 'generation' was not significant. The absence of changes between the F1 and F2 generations can be attributed to study limitations. Skinner et al. [73] demonstrated that the administration of 0.25 mg.kg^−1^ of DDT to pregnant rats (parental generation) induces a transgenerational effect, resulting in weight gain and metabolic syndrome starting from the third descendant generation, which was not directly exposed to the organochlorine. Thus, the lack of changes between the first and second generations does not rule out the possibility of alterations in subsequent generations due to DDT exposure.

In the analysis of the hypothetical model without the 'generation' factor, a good model fit was observed, with the new proposal revealing deleterious tissue responses in the groups exposed to DDT residues, with effects especially related to sex and the developmental periods of the animals. Thus, the following discussion will address the latent variables summarized by the principal component analysis, as well as their associations and relationships analyzed in the model evaluated in the confirmatory factor analysis.

### 4.1 Hepatic and renal tissues

In the hepatic tissue, it was possible to verify that exposure in the order of DDD < DDE < DDD/DDE, especially in females at the beginning of the pubertal period (PND35), promoted an increase in the ‘Activity of hepatic enzymes GR, CAT, GPT, and BuChE’, without significant relationships with ‘Hepatic oxidative damage’. This increase in enzymatic activity, especially in females, may be related to the elevation of the activity of other hepatic microsomal enzymes, such as cytochrome P450 isoforms, induced by exposure to DDT and its residues, as already demonstrated by Fox et al. [23], Harada et al. [27], Motohira et al. [54], Nims et al. [57], Sierra-Santoyo et al. [71]. Sierra-Santoyo and collaborators (2000) revealed that the exposure of Wistar rats to DDT resulted in the induction of cytochrome P450 isoform, especially 3A2 and 3A1, in contrast to what was observed in males, where the induction was considerably lower. Other isoforms, like 2B1 and 2B2, are induced similarly in both males and females [51], [57], [71].

The oxidation-reduction processes catalyzed by cytochrome P450 isoforms are naturally sources of ROS, and an equilibrium between production and elimination is required to avoid damage to cellular components such as lipids, proteins, and DNA [37], [50], [51], [52], [64], [82]. Migliaccio and collaborators (2019b) propose that, in response to the stimulus of cytochrome P450 isoforms generated by DDE exposure, the mitochondria play a central role in oxidative balance, with an increase in the synthesis of uncoupling proteins to reduce ROS production [50], [51].

Therefore, even though DDD and DDE did not induce a process of lipoperoxidation, the increase in antioxidant activity during the pubertal period demonstrated that exposure to DDT residues, especially in females, promoted an increase in ROS, necessitating a higher activity of antioxidant enzymes, such as CAT, to neutralize hydrogen peroxide (H_2_O_2_), and GR, to regenerate reduced glutathione, as well as stimulating mitochondrial oxidative uncoupling to reduce ROS production.

The enzymes BuChE and ALT were evaluated due to their relevance as markers of hepatic damage. BuChE, synthesized in the liver and linked to albumin production, was included by Khan et al. [35] as an indicator of liver cell synthesis. According to Lampón et al. [39], a low-grade inflammatory process is associated with an increase in inflammatory proteins, which correlates with elevated BuChE activity. The rise in BuChE observed in offspring at PND35 may be a potential precursor to metabolic syndrome, as also reported by Randell et al. [63]. Regarding ALT, increased levels of hepatic transaminases after exposure to DDD and DDE had been identified in a previous study [83]. Abnormal ALT levels may reflect high levels of hepatic amino acid transamination, prior to the development of hepatic steatosis and metabolic syndrome [75]. Thus, the increase in both enzymes, BuChE and ALT, may be related to an increased risk of developing metabolic syndrome and low-grade hepatic inflammation, even in the absence of hepatic damage.

In the renal tissue, exposure to DDD and DDE, especially in females at the onset of puberty (PND35), showed inverse relationships with ‘Renal Antioxidant Activity’ and ‘Renal Lipoperoxidation/GST’. GST plays an important role in the detoxification of xenobiotics [47]. Therefore, the increase in GST in the renal tissue may be related to a higher rate of conjugation via reduced glutathione [20], [4], [47]. This stimulation, especially at the onset of puberty, was in line with the changes described for hepatic tissue, demonstrating the need for increased glutathione conjugation to prevent potential tissue damage arising from oxidative stress, as measured here by LPO.

The results in the animals at PND105 indicate that the changes observed at PND35 were not continuous into adulthood. Although some adult animals showed morphological changes, such as benign cysts in the liver (Fig. 3) and urinary tract stones (Fig. 4). However, no biochemical changes were observed in animals exposed to DDD and DDE at PND105. This suggests that animals may be more susceptible to trace concentrations of DDD and DDE during puberty.

### 4.2 Brain tissue

In the brain tissue, the synergy between DDD and DDE, in females at the onset of puberty, led to a reduction in the activity of ‘Brain Acetylcholinesterase’ and ‘Brain Tissue Mass’. The brain tissue is a target organ in DDT intoxication and its residues [11]. Tomiyama et al. [78] emphasized that following exposure to DDT, DDD, or DDE, the main metabolite that crosses the blood-brain barrier and reaches brain tissue is DDE.

A reduction in ‘brain tissue mass’ was observed early in puberty, especially in females, suggesting a possible delay in brain development. Studies indicate that brain development is dependent on sex and the duration of exposure to endocrine disruptors, such as DDE [65]. The brain is an organ highly dependent on hormonal signaling for its development, and for this reason, the endocrine-disrupting effects caused by these compounds should be considered when analyzing this tissue [26]. The chemical similarity of DDD and DDE with endogenous hormonal molecules facilitates their binding to steroidal receptors, modulating signaling through estrogen, androgen, and thyroid receptors [68], [77]. The bioactivity in androgenic and estrogenic receptors was defined by the ToxCast Pathway Model and shows estrogenic agonist activity in the order DDT (AUC=19 %) > DDD (AUC=7.3 %) > DDE (AUC=6.8 %) and androgenic antagonist activity in the order DDE (AUC=25 %) > DDD (AUC=9.7 %) > DDT (AUC=6.4 %) [79], [81], [80].

The reduced AChE activity and brain mass may result from stimulation of the central nervous system (CNS), which alters the flow of Na+ /K+ ions across the axonal membrane by keeping sodium channels open longer, leading to hyperexcitability. This could be a triggering stimulus for subsequent neurotoxic effects, as observed by Costa [11] and Dardiotis et al. [14]. Although neurological disorders were not evaluated in the animals, neurodegenerative and neurobehavioral damage is expected when there is long-term exposure to DDT and its residues [14], [28].

According to Fonseca et al. [22], in rats exposed for two months to DDT (400 µg of DDT.g^−1^ of food), there was a 30 % decrease in muscarinic receptors in the cerebellum and a higher affinity for the cerebral cortex. It is plausible to propose the reduction of muscarinic receptors in the present study, as it aligns with findings in the cholinergic system, showing lower activity of the enzyme AChE, especially in females at PND35, exposed to the synergy of DDD and DDE. A reduction in AChE activity was also identified in other studies, which indicate a relationship between DDE exposure and decreased enzyme activity [69], [84].

These findings support the hypothesis of a possible delay in the development of brain tissue, particularly related to endocrine dysregulation caused by DDD and DDE in females.

### 4.3 White adipose tissue (WAT) and biometric development

The results suggest that exposure to DDD and DDE in adulthood, especially in females, caused disturbances in the WAT, resulting in an increase in the mass in visceral and perigonadal WAT, as well as intensifying the process of LPO in the perigonadal fraction. WAT is the main tissue for storing DDT residues, with storage increasing in the order of DDD < DDE. This accumulation is particularly notable due to the reduced metabolism of DDE, resulting in prolonged storage of this form of the residue [53], [55], [74]. Additionally, since the biotransformation of DDD leads to the formation of DDE [4], a higher storage rate of DDE is expected when combined with DDD.

This increase in adipocyte mass may occur through two processes, hypertrophy or hyperplasia [15]. An *in vitro* study with pre-adipocytes (3T3-L1 adipocytes) showed that DDE contributes to increased adipogenesis [36], which implies an accentuated accumulation of DDE in WAT [13], [40]. In this study, the increase in adipocyte mass was associated with lipoperoxidation in the perigonadal fraction of WAT, suggesting that DDE accumulation may affect both the size and number of adipocytes, as well as contribute to oxidative stress in WAT.

Studies indicate that the oxidative changes accompanying adipose tissue expansion in processes like adipogenesis may precede adipocyte dysfunction and contribute to the development of metabolic disorders [29]. With the increase in adipocyte mass, it is expected that oxidative stress will develop in the WAT [15], [29], [42], [56]. Which becomes evident with the increase in tissue lipoperoxidation levels. The most noticeable response in females is in line with the study by Lugo et al. [42], which identified a positive association between lipid accumulation and lipoperoxidation levels in both sexes, with a higher susceptibility in females.

In addition to the findings in WAT, an obesogenic effect was observed, noticeable with the ‘Weight Gain’ and increased ‘Lee Index’, especially in adult females exposed to DDD and DDE. The positive association between the obesogenic effect and age can be explained by the higher body fat content in the animals during adulthood, naturally contributing to the greater deposition of DDE in adipocytes [13].

Regarding sex-related differences, although the hypothetical model identified a higher susceptibility to the obesogenic effect in females, it is important to note that this effect was also present in males, consistent with a previous study in which males exposed for 70 days to trace concentrations of DDD and DDE showed an increase in body weight and the Lee index [83].

In females, the results align with other studies that show a greater susceptibility in females, suggesting that weight gain is stimulated by DDE exposure during the intrauterine period [32], [38], [61]. Cano-Sancho et al. [7] identified a moderate positive association between prenatal DDE exposure and increased adipose mass in adulthood. Therefore, the combined effects on WAT and biometric development suggest a possible obesogenic effect in adult animals exposed to the DDD/DDE combination, which may act as a precursor to future metabolic disorders.

## 5 Conclusion

The synergy between DDD and DDE, along with the influence of sex, especially in females, was linked to the effects observed across all analyzed tissues. Daily doses 10-fold below the LOAEL of p,p’-DDD and 3,000-fold below the LOAEL of p,p’-DDE were sufficient to disrupt hepatic, renal, and cerebral tissues at the onset of puberty. Furthermore, the DDD/DDE combination contributed to changes in adipocyte mass and biometric development in adult animals, suggesting a potential obesogenic effect.

## Funding sources

This study was financed in part by: Coordination for the Improvement of Higher Education Personnel (10.13039/501100002322CAPES), Brazil; National Council for Scientific and Technological Development (10.13039/501100003593CNPq), Brazil; The Global Challenge Research Fund of Wales (10.13039/100015689GCRF - Code JA1910RD12JA1910RD12), United Kingdom.

## Authorship statement

All persons who meet authorship criteria are listed as authors, and all authors certify that they have participated sufficiently in the work to take public responsibility for the content, including participation in the concept, design, acquisition, analysis, interpretation of data, writing, revision of the manuscript and approved the final version to be published. All authors agree to be responsible for all aspects of the manuscript, ensuring that issues related to the accuracy or completeness of any part of the manuscript are appropriately investigated and resolved. Furthermore, each author certifies that this material or similar material has not been and will not be submitted to or published in any other publication before its appearance in the *Toxicology Reports*.

All persons who have made substantial contributions to the work reported in the manuscript, including those who provided editing and writing assistance but who are not authors, are named in the Acknowledgments section of the manuscript and have given their written permission to be named. If the manuscript does not include Acknowledgments, it is because the authors have not received substantial contributions from nonauthors.

## CRediT authorship contribution statement

**Guimarães Ana Tereza Bittencourt:** Writing – review & editing, Writing – original draft, Resources, Project administration, Formal analysis, Conceptualization. **Marek Carla Brugin:** Resources, Methodology, Conceptualization. **Pavlak Jaine Luana:** Investigation, Data curation. **Azevedo Camilla de Marchi Sanches:** Methodology, Investigation, Data curation. **Coleraus Fernanda:** Writing – review & editing, Writing – original draft, Supervision, Methodology, Investigation, Data curation, Conceptualization.

## Declaration of Generative AI and AI-assisted technologies in the writing process

During the preparation of this work, the author used OpenAI's ChatGPT to improve the readability and language of the manuscript after its translation into English. After using this tool, the author reviewed and edited the content as needed and takes full responsibility for the content of the publication.

## Declaration of Competing Interest

The authors declare the following financial interests/personal relationships which may be considered as potential competing interests: Ana Tereza Bittencourt Guimaraes reports financial support was provided by Global Challenge Research Fund of Wales. Ana Terezqa Bittencourt Guimaraes reports a relationship with National Council for Scientific and Technological Development that includes: funding grants. If there are other authors, they declare that they have no known competing financial interests or personal relationships that could have appeared to influence the work reported in this paper.

## Data Availability

Data will be made available on request.

## References

[1] Achour A., Derouiche A., Barhoumi B., Kort B., Cherif D., Bouabdallah S. (2017). Organochlorine pesticides and polychlorinated biphenyls in human adipose tissue from northern Tunisia: current extent of contamination and contributions of socio-demographic characteristics and dietary habits. Environ. Res..

[2] H. AebiIn: Bergmeyer, H.U. editor. Methods of enzymatic analysis. 2th edition. New York: (1974), v. 2.

[3] Agarwal A., Gupta S., Sharma R.K. (2005). Role of oxidative stress in female reproduction. Reprod. Biol. Endocrinol..

[4] ATSDR. Toxicological Profile for DDT, DDE and DDD. vol. 2022. Atlanta, GA: U.S. Department of Health and Human Services; 2022. http://dx.doi.org/〈10.1155/2013/286524〉.

[5] Belda M.P., González-Franco J.A., Rubio R., Campillo N., Hernández-Cordoba M., Torres C. (2021). Occurrence of organochlorine pesticides in human tissues assessed using a microextraction procedure and gas chromatography-mass spectrometry. J. Anal. Toxicol..

[6] Bradford M. (1976). A rapid and sensitive method for the quantification of microgram quantities of protein utilizing the principle of protein-dye binding. Anal. Biochem..

[7] Cano-Sancho G., Salmon A.G., Merrill. Obesity M.A. (2017). Integrated Systematic Review and Meta-Analysis. Environ Health Perspect.

[8] Chávez-Almazán L.A., Saldarriaga-Noreña H.A., Díaz-González L., Garibo-Ruiz D., Waliszewski S.M. (2023). Relationship between DDT concentrations with multiparity and breastfeeding history. Bull. Environ. Contam. Toxicol..

[9] J.C. Chen, B.O. Baumert, Y. Li, Y. Li, S. Pan, S. Robinson, B. Rubbo, E. Costello, J. He, H. Hampson, E. Beglarian, S. Rock, J. A. Goodrich, S. P. Eckel, M.T. Aung, R. McConnell, D. V. Conti, L. Chatzi. Associations of per- and polyfluoroalkyl substances, polychlorinated biphenyls, organochlorine pesticides, and polybrominated diphenyl ethers with oxidative stress markers: A systematic review and meta-analysis, Environmental Research, 239 (1) (2023), doi: 10.1016/j.envres.2023.117308. 10.1016/j.envres.2023.117308 PMC10841434 37813138

[10] Chiu W.A., Axelrad D.A., Dalaijamts C., Dockins C., Shao K., Shapiro A.J. (2018). Beyond the RfD: Broad application of a probabilistic approach to improve chemical dose–response assessments for noncancer effects. Environ. Health Perspect..

[11] Costa L.G. (2015). The Neurotoxicity of Organochlorine and Pyrethroid Pesticides.

[12] Crouch R.K., Gandy S.C., Kimsey G. (1981). The inhibition of islet superoxide dismutase by diabetogenic drugs. Diabetes.

[13] Dandridge Frugé A., Cases M.G., Schildkraut J.M., Demark-Wahnefried W., Dandridge A. (2016). Associations between obesity, body fat distribution, weight loss and weight cycling on serum pesticide concentrations. HHS Public Access.

[14] Dardiotis E., Aloizou A.M., Sakalakis E., Siokas V., Koureas M., Xiromerisiou G. (2020). Organochlorine pesticide levels in Greek patients with Parkinson’s disease. Toxicol. Rep..

[15] De Fano M., Bartolini D., Tortoioli C., Vermigli C., Malara M., Galli F. (2022). Adipose tissue plasticity in response to pathophysiological cues: a connecting link between obesity and its associated Comorbidities. Int J. Mol. Sci..

[16] Draper H.H., Hadley M. (1990). Malondialdehyde determination as index of lipid peroxidation. Methods Enzym..

[17] G.L. Ellman, K.D. Courtney, V. Andres, R.M. Featherstone. A new and rapid colorimetric determination of acetylcholinesterase activity. Biochemical Pharmacology, vol. 7, n^o^ 2, p. 88–95, 1961. 10.1016/0006-2952(61)90145-9. 13726518

[18] Elmore S.E., La Merrill M.A. (2019). Oxidative phosphorylation impairment by DDT and DDE. Front Endocrinol..

[19] Eriksson P., Nilsson-Håkansson L., Nordberg A., Aspberg A., Fredriksson A. (1990). Neonatal exposure to DDT and its fatty acid conjugate: effects on cholinergic and behavioural variables in the adult mouse. Neurotoxicology.

[20] Espinosa-Diez C., Miguel V., Mennerich D., Kietzmann T., Sánchez-Pérez P., Cadenas S. (2015). Antioxidant responses and cellular adjustments to oxidative stress. Redox Biol..

[21] Fernandes C.L.F., Volcaõ L.M., Ramires P.F., Moura R.R., De, Da Silva Júnior F.M.R. (2020). Distribution of pesticides in agricultural and urban soils of Brazil: a critical review. Environ. Sci. Process Impacts.

[22] Fonseca M.I., Aguilar J.S., López C., García Fernández J.C., De Robertis E. (1986). Regional effect of organochlorine insecticides on cholinergic muscarinic receptors of rat brain. Toxicol. Appl. Pharm..

[23] Fox S.D., Roman J.M., Issaq H.J., Nims R.W. (1998). Metabolic conversion of 1,1-dichloro-2,2-bis(p-chlorophenyl)ethane (DDD) to 1,1-dichloro-2,2-bis(p-chlorophenyl)ethylene (DDE) in the male F344/NCr Rat. Arch. Environ. Contam. Toxicol..

[24] Ghosh R., Siddarth M., Singh N., Tyagi V., Kare P.K., Banerjee B.D. (2017). Organochlorine pesticide level in patients with chronic kidney disease of unknown etiology and its association with renal function. Environ. Health Prev. Med.

[25] Gold B., Brunk G. (1982). Metabolism of 1,1,1-trichloro-2,2-bis(p-chlorophenyl)ethane and 1,1-dichloro-2,2-bis(p-chlorophenyl)ethane in the mouse. Chem. Biol. Inter..

[26] Grossklaus R., Liesenkötter K.P., Doubek K., Völzke H., Gaertner R. (2023). Iodine deficiency, maternal hypothyroxinemia and endocrine disrupters affecting fetal brain development: a scoping review. Nutrients.

[27] Harada T., Takeda M., Kojima S., Tomiyama N. (2016). Toxicity and carcinogenicity of dichlorodiphenyltrichloroethane (DDT). Toxicol. Res..

[28] Hawkey A.B., Glazer L., Dean C., Wells C.N., Odamah K.-A., Slotkin T.A. (2020). Adult exposure to insecticides causes persistent behavioral and neurochemical alterations in zebrafish. Neurotoxicol Teratol..

[29] Jankovic A., Korac A., Buzadzic B., Otasevic V., Stancic A., Daiber A. (2015). Redox implications in adipose tissue (dys)function-a new look at old acquaintances. Redox Biol..

[30] Jawaid A., Jehle K., Mansuy I.M. (2020). Impact of parental exposure on offspring health in humans. Trends Genet..

[31] Kao C.C., Que D.E., Bongo S.J., Tayo L.L., Lin Y.H., Lin C.W. (2019). Residue levels of organochlorine pesticides in breast milk and its associations with cord blood thyroid hormones and the offspring’s neurodevelopment. Int J. Environ. Res. Public Health.

[32] Karmaus W., Osuch J.R., Eneli I., Mudd L.M., Zhang J., Mikucki D. (2009). Maternal levels of dichlorodiphenyl-dichloroethylene (DDE) may increase weight and body mass index in adult female offspring. Occup. Environ. Med.

[33] Keen J.H., Habig W.H., Jakoby W.B. (1976). Mechanism for several activities of the gluthatione S- transferases. J. Biol. Chem..

[34] Kesic R., Elliott J.E., Fremlin K.M., Gauthier L., Drouillard K.G., Bishop C.A. (2021). Continuing persistence and biomagnification of DDT and metabolites in northern temperate fruit orchard avian food chains. Environ. Toxicol. Chem..

[35] Khan M.G. (1962). The evaluation of serum pseudocholinesterase as a liver function test. Ulst. Med. J..

[36] Kim J., Sun Q., Yue Y., Yoon K.S., Whang K.Y., Marshall Clark J. (2016). 4,4’-Dichlorodiphenyltrichloroethane (DDT) and 4,4’-dichlorodiphenyldichloroethylene (DDE) promote adipogenesis in 3T3-L1 adipocyte cell culture. Pest. Biochem. Physiol..

[37] Kitamura S., Shimizu Y., Shiraga Y., Yoshida M., Sugihara K., Ohta S. (2002). Reductive metabolism of p,p-DDT and o,p-DDT by rat liver cytochrome P450 abstract. Drug Metab. Dispos..

[38] La Merrill M.A., Krigbaum N.Y., Cirillo P.M., Cohn B.A. (2020). Association between maternal exposure to the pesticide dichlorodiphenyltrichloroethane (DDT) and risk of obesity in middle age. Int. J. Obes..

[39] Lampón N., Hermida-Cadahia E.F., Riveiro A., Tutor Carlos (2012). Association between butyrylcholinesterase activity and low-grade systemic inflammation. Ann. Hepatol..

[40] Lee D.H., Jacobs D.R., Park H.Y., Carpenter D.O. (2017). A role of low dose chemical mixtures in adipose tissue in carcinogenesis. Environ. Int..

[41] Little T.D. (2013). Longitudinal Structural Equation Modeling: Methodology in the Social Sciences.

[42] Lugo R., Avila-Nava A., Pech-Aguilar A.G., Medina-Vera I., Guevara-Cruz M., Gutiérrez Solis A.L. (2022). Relationship between lipid accumulation product and oxidative biomarkers by gender in adults from Yucatan, Mexico. Sci. Rep..

[43] Makgoba L., Abrams A., Röösli M., Cissé G., Dalvie M.A. (2024). DDT contamination in water resources of some African countries and its impact on water quality and human health. Heliyon.

[44] Mansouri A., Cregut M., Abbes C., Durand M.J., Landoulsi A., Thouand G. (2017). The environmental issues of DDT pollution and bioremediation: a multidisciplinary review. Appl. Biochem. Biotechnol..

[45] F. K. Marcondes, F. J. Bianchi, A. P. Tanno. Determination of the estrous cycle phases of rats: some helpful considerations. Braz. J. Biol. 62 (42a) (2002), doi:10.1590/S1519-69842002000400008. 10.1590/s1519-69842002000400008 12659010

[46] Marouani N., Hallegue D., Sakly M., Benkhalifa M., Rhouma K.B., Tebourbi O. (2017). Involvement of oxidative stress in the mechanism of p,p’-DDT-induced nephrotoxicity in adult rats. Gen. Physiol. Biophys..

[47] Mazari A.M.A., Zhang L., Ye Z.W., Zhang J., Tew K.D., Townsend D.M. (2023). The multifaceted role of glutathione S-transferases in health and disease. Biomolecules.

[48] Melnick R.L. (2002). Carcinogenicity and mechanistic insights on the behavior of epoxides and epoxide-forming chemicals. Ann. N Y Acad. Sci..

[49] Meyer J.N., Hartman J.H., Mello D.F. (2018). Mitochondrial toxicity. Toxicol. Sci..

[50] Migliaccio V., Di Gregorio I., Putti R., Lionetti L. (2019). Mitochondrial involvement in the adaptive response to chronic exposure to environmental pollutants and high-fat feeding in a rat liver and testis. Cells.

[51] Migliaccio V., Scudiero R., Sica R., Lionetti L., Putti R. (2019). Oxidative stress and mitochondrial uncoupling protein 2 expression in hepatic steatosis induced by exposure to xenobiotic DDE and high fat diet in male Wistar rats. PLoS One.

[52] Morales-Prieto N., Abril N. (2017). REDOX proteomics reveals energy metabolism alterations in the liver of M. spretus mice exposed to p, p′-DDE. Chemosphere.

[53] Morgan D.P., Roan C.C. (1971). Absorption, storage, and metabolic conversion of ingested ddt and ddt metabolites in man. Arch. Environ. Health.

[54] Motohira K., Yohannes Y.B., Ikenaka Y., Eguchi A., Nakayama S.M.M., Wepener V. (2023). Investigation of dichlorodiphenyltrichloroethane (DDT) on xenobiotic enzyme disruption and metabolomic bile acid biosynthesis in DDT-sprayed areas using wild rats. J. Vet. Med. Sci..

[55] Mühlebach S., Moor M.J., Wyss P.A., Bickel M.H. (1991). Kinetics of distribution and elimination of DDE in rats. Xenobiotica.

[56] Murdolo G., Piroddi M., Luchetti F., Tortoioli C., Canonico B., Zerbinati C. (2013). Oxidative stress and lipid peroxidation by-products at the crossroad between adipose organ dysregulation and obesity-linked insulin resistance. Biochimie.

[57] Nims R.W., Lubet R.A., Fox S.D., Jones C.R., Thomas P.E., Reddy A.B. (1998). Comparative pharmacodynamics of cyP2B induction BY DDT, DDE, AND DDD In male rat liver and cultured rat hepatocytes. J. Toxicol. Environ. Health A.

[58] Pérez-Maldonado I.N., Athanasiadou M., Yáñez L., González-Amaro R., Bergman A., Díaz-Barriga F. (2006). DDE-induced apoptosis in children exposed to the DDT metabolite. Sci. Total Environ..

[59] Pérez-Maldonado I.N., Herrera C., Batres L.E., González-Amaro R., Díaz-Barriga F., Yáñez L. (2005). DDT-induced oxidative damage in human blood mononuclear cells. Environ. Res.

[60] Pestana D., Teixeira D., Meireles M., Marques C., Norberto S., Sá C. (2017). Adipose tissue dysfunction as a central mechanism leading to dysmetabolic obesity triggered by chronic exposure to p,p’-DDE. Sci. Rep..

[61] Plouffe L., Bosson-Rieutort D., Madaniyazi L., Iwai-Shimada M., Nakai K., Tatsuta N. (2020). Estimated postnatal p,p’-DDT and p,p’-DDE levels and body mass index at 42 months of age in a longitudinal study of Japanese children. Environ. Health.

[62] R Core Team, : A Language and Environment for Statistical Computing, R Foundation for Statistical Computing, Vienna, Austria., 2020. <https://www.R-project.org/>.

[63] Randell E.W., Mathews M.S., Zhang H., Seraj J.S., Sun G. (2005). Relationship between serum butyrylcholinesterase and the metabolic syndrome. Clin. Biochem..

[64] Rashba-Step J., Cederbaum A.I. (1994). Generation of reactive oxygen intermediates by human liver microsomes in the presence of NADPH or NADH. Mol. Pharm..

[65] Rebuli M.E., Patisaul H.B. (2016). Assessment of sex specific endocrine disrupting effects in the prenatal and pre-pubertal rodent brain. J. Steroid Biochem. Mol. Biol..

[66] Richardson J.R., Fitsanakis V., Westerink R.H.S., Kanthasamy A.G. (2019). Neurotoxicity of pesticides. Acta Neuropathol..

[67] Rodríguez-Alcalá L.M., Sá C., Pimentel L.L., Pestana D., Teixeira D., Faria A. (2015). Endocrine disruptor DDE associated with a high-fat diet enhances the impairment of liver fatty acid composition in rats. J. Agric. Food Chem..

[68] Safe S. (2020). Recent advances in understanding endocrine disruptors: DDT and related compounds. Fac. Rev..

[69] Samareh, Asadikaram A., MojtabaAbbasi-Jorjandi G. (2022). Occupational exposure to pesticides in farmworkers and the oxidative markers. Toxicol. Ind. Health.

[70] Seo H., Little T.D., Shogren K.A. (2016). On the benefits of latent variable modeling for norming scales: The case of the Supports Intensity Scale – Children’s Version. International Journal of Behavioral Development.

[71] Sierra-Santoyo A., Herńandez M., Albores A., Cebrián M.E. (2000). Sex-dependent regulation of hepatic cytochrome P-450 by DDT. Toxicol. Sci..

[72] Sies H., Koch O.R., Martino E., Boveris A. Aumento da liberação biliar de glutationa dissulfeto em ratos cronicamente tratados com etanol. FEBS Lett. 1979 15 de julho; 103(2):287-90. PMID: 467672. 10.1016/0014-5793(79)81346-0. 467672

[73] Skinner M.K., Manikkam M., Tracey R., Guerrero-Bosagna C., Haque M. (2013). Ancestral dichlorodiphenyltrichloroethane (DDT) exposure promotes epigenetic transgenerational inheritance of obesity. BMC Med.

[74] A.G. SmithToxicology of DDT and Some Analogues. Third Edit. Elsevier Inc.; (2010), vol. 2http://10.1016/B978-0-12-374367-1.00093-8.

[75] Sookoian S., Pirola C.J. (2012). Alanine and aspartate aminotransferase and glutamine-cycling pathway: Their roles in pathogenesis of metabolic syndrome. World J. Gastroenterol..

[76] Stockholm Convention. Stockholm Convention on Persistent Organic Pollutants (POPs). Revised in 2018. Secretariat of the Stockholm Convention 2018. 10.1351/goldbook.s06019.

[77] Timokhina E.P., Yaglov V.V., Nazimova S.V. (2021). Dichlorodiphenyltrichloroethane and the adrenal gland: from toxicity to endocrine disruption. Toxics.

[78] Tomiyama N., Watanabe M., Takeda M., Harada T., Kobayashi H. (2003). A comparative study on the reliablility of toxicokinetic parameters for predicting hepatotoxicity of ddt in rats receiving a single or repeated administration.

[79] U.S. Environmental Protection Agency. Comptox Chemicals Dashboard. https://comptox.epa.gov/dashboard/chemical/details/DTXSID4020373 (accessed March 28, 2025).

[80] U.S. Environmental Protection Agency. Comptox Chemicals Dashboard. https://comptox.epa.gov/dashboard/chemical/details/DTXSID4020375 (accessed March 28, 2025).

[81] U.S. Environmental Protection Agency. Comptox Chemicals Dashboard. https://comptox.epa.gov/dashboard/chemical/details/DTXSID9020374 (accessed March 28, 2025).

[82] VoPham T., Bertrand K.A., Hart J.E., Laden F., Brooks M.M., Yuan J.M. (2017). Pesticide exposure and liver cancer: a review. Cancer Causes Control.

[83] Wolfart J.C., Theodoro J.L., Silva F.C., de Oliveira C.M.R., Ferreira N.G.C., Bittencourt Guimarães A.T. (2023). Metabolic consequences of the water we drink: a study based on field evidence and animal model experimentation. Toxics.

[84] Yousefi F., Asadikaram G., Karamouzian S. (2022). Organochlorine and organophosphorus pesticides may induce brain cancer through oxidative stress. Toxicol. Ind. Health.

