# Supplementary material for: Multigenerational exposure to trace concentrations of DDT residues in Wistar rats: Effects on biometric development and biochemical parameters
Source: Toxicol Rep. 2025 Mar 26;14:102012. doi: 10.1016/j.toxrep.2025.102012 (PMC11997410; doi:10.1016/j.toxrep.2025.102012)
Supplement: Supplementary file 1 — Supplementary material [file mmc1.docx]

**Supplementary Materials**

**Table S1. Biometric analysis of female Wistar rats from the first (F1) and second (F2) generations exposed to residues of DDT.**

| Groups | | | **WGR** | **ΔAC** | **ΔNAL** | **LI** | **SI**  **Brain** | **SI**  **Liver** | **SI**  **Kidney** | **SI**  **VAT** | **SI**  **PAT** | **FC_Total_** | **WC_Total_** | **WGTIR** |
| --- | --- | --- | --- | --- | --- | --- | --- | --- | --- | --- | --- | --- | --- | --- |
| Generation F1 | PND35 | CTL | 1.74 ± 0.05 | 2.50 ± 0.15 | 4.54 ± 0.27 | 326.1 ± 3.7 | 1.63 ± 0.07 | 4.11 ± 0.09 | 0.45 ± 0.06 | 0.08 ± 0.01 | 0.16 ± 0.01 | 155 ± 10 | 323 ± 14 | 1.49 ± 0.12 |
|  |  | DDD | 1.59 ± 0.07 | 2.35 ± 0.27 | 4.17 ± 0.33 | 332.1 ± 6.7 | 1.58 ± 0.06 | 4.00 ± 0.13 | 0.48 ± 0.01 | 0.09 ± 0.02 | 0.21 ± 0.01 | 191 ± 9 | 364 ± 17 | 1.26 ± 0.13 |
|  |  | DDE | 1.55 ± 0.06 | 2.25 ± 0.19 | 4.14 ± 0.38 | 343.3 ± 10.9 | 1.55 ± 0.04 | 4.04 ± 0.11 | 0.47 ± 0.01 | 0.11 ± 0.00 | 0.22 ± 0.00 | 165 ± 12 | 305 ± 10 | 1.38 ± 0.06 |
|  |  | DDD/DDE | 1.75 ± 0.12 | 2.75 ± 0.27 | 4.52 ± 0.36 | 336.6 ± 7.7 | 1.51 ± 0.05 | 4.14 ± 0.10 | 0.50 ± 0.01 | 0.11 ± 0.02 | 0.21 ± 0.02 | 139 ± 5 | 318 ± 20 | 1.81 ± 0.05 |
|  | PND105 | CTL | 5.04 ± 0.15 | 6.21 ± 0.18 | 9.58 ± 0.29 | 317.9 ± 3.6 | 0.78 ± 0.01 | 3.50 ± 0.11 | 0.37 ± 0.03 | 1.59 ± 0.38 | 1.08 ± 0.18 | 1344 ± 34 | 2663 ± 45 | 0.53 ± 0.01 |
|  |  | DDD | 4.97 ± 0.25 | 5.68 ± 0.38 | 9.47 ± 0.25 | 317.0 ± 3.7 | 0.79 ± 0.02 | 3.21 ± 0.15 | 0.38 ± 0.01 | 1.79 ± 0.29 | 1.21 ± 0.20 | 1366 ± 31 | 2543 ± 81 | 0.52 ± 0.00 |
|  |  | DDE | 5.23 ± 0.32 | 5.24 ± 0.29 | 9.60 ± 0.40 | 322.6 ± 1.6 | 0.78 ± 0.03 | 3.51 ± 0.15 | 0.38 ± 0.01 | 1.69 ± 0.31 | 1.09 ± 0.16 | 1279 ± 34 | 2298 ± 28 | 0.57 ± 0.01 |
|  |  | DDD/DDE | 4.98 ± 0.25 | 5.75 ± 0.33 | 9.27 ± 0.26 | 328.1 ± 4.2 | 0.78 ± 0.01 | 3.40 ± 0.05 | 0.41 ± 0.00 | 1.75 ± 0.37 | 1.51 ± 0.25 | 1438 ± 39 | 3202 ± 63 | 0.51 ± 0.01 |
| Generation F2 | PND35 | CTL | 1.51 ± 0.12 | 2.05 ± 0.22 | 3.87 ± 0.15 | 348.3 ± 5.0 | 1.60 ± 0.07 | 4.08 ± 0.13 | 0.50 ± 0.01 | 0.17 ± 0.03 | 0.18 ± 0.04 | 141 ± 11 | 234 ± 16 | 1.59 ± 0.02 |
|  |  | DDD | 1.45 ± 0.12 | 1.95 ± 0.30 | 3.88 ± 0.36 | 350.6 ± 3.5 | 1.66 ± 0.05 | 4.27 ± 0.13 | 0.49 ± 0.01 | 0.13 ± 0.03 | 0.19 ± 0.02 | 153 ± 10 | 218 ± 13 | 1.38 ± 0.05 |
|  |  | DDE | 1.52 ± 0.18 | 2.54 ± 0.25 | 3.82 ± 0.25 | 342.5 ± 9.7 | 1.65 ± 0.07 | 4.07 ± 0.12 | 0.46 ± 0.01 | 0.11 ± 0.01 | 0.18 ± 0.01 | 139 ± 10 | 244 ± 18 | 1.50 ± 0.07 |
|  |  | DDD/DDE | 1.40 ± 0.12 | 1.97 ± 0.32 | 3.51 ± 0.33 | 353.6 ± 4.0 | 1.27 ± 0.22 | 4.21 ± 0.05 | 0.51 ± 0.01 | 0.14 ± 0.02 | 0.25 ± 0.03 | 123 ± 9 | 196 ± 13 | 1.91 ± 0.05 |
|  | PND105 | CTL | 5.03 ± 0.14 | 5.78 ± 0.24 | 8.92 ± 0.26 | 329.5 ± 5.6 | 0.80 ± 0.01 | 3.33 ± 0.06 | 0.35 ± 0.00 | 1.55 ± 0.28 | 0.98 ± 0.11 | 1359 ± 31 | 2682 ± 75 | 0.52 ± 0.01 |
|  |  | DDD | 4.80 ± 0.19 | 5.37 ± 0.18 | 8.90 ± 0.32 | 323.4 ± 4.9 | 0.80 ± 0.02 | 3.35 ± 0.12 | 0.39 ± 0.01 | 1.60 ± 0.25 | 1.15 ± 0.17 | 1322 ± 16 | 2755 ± 12 | 0.52 ± 0.02 |
|  |  | DDE | 4.80 ± 0.31 | 6.01 ± 0.31 | 8.81 ± 0.37 | 330.7 ± 3.8 | 0.81 ± 0.01 | 3.11 ± 0.09 | 0.35 ± 0.01 | 1.45 ± 0.19 | 0.81 ± 0.05 | 1271 ± 2 | 2575 ± 17 | 0.53 ± 0.00 |
|  |  | DDD/DDE | 5.00 ± 0.54 | 5.69 ± 0.39 | 9.14 ± 0.37 | 324.3 ± 4.0 | 0.80 ± 0.01 | 3.26 ± 0.11 | 0.36 ± 0.00 | 1.51 ± 0.16 | 1.09 ± 0.16 | 1353 ± 24 | 2615 ± 64 | 0.51 ± 0.01 |
| Generation | | | 0.1739 | 0.2281 | 0.001 | 0.0008 | 0.9352 | 0.6611 | 0.764 | 0.5083 | 0.0893 | 0.0158 | 0.0036 | 0.0682 |
| Age | | | < 0.0001 | < 0.0001 | < 0.0001 | < 0.0001 | < 0.0001 | < 0.0001 | < 0.0001 | < 0.0001 | < 0.0001 | < 0.0001 | < 0.0001 | < 0.0001 |
| Group | | | 0.9024 | 0.518 | 0.9408 | 0.4462 | 0.0437 | 0.78 | 0.2032 | 0.9702 | 0.1355 | 0.0081 | < 0.0001 | < 0.0001 |
| Generation * Age | | | 0.8721 | 0.262 | 0.9039 | 0.1332 | 0.5456 | 0.0457 | 0.1089 | 0.2969 | 0.1118 | 0.756 | 0.0447 | 0.0472 |
| Generation * Group | | | 0.9912 | 0.0601 | 0.9677 | 0.3738 | 0.2881 | 0.0916 | 0.4863 | 0.9604 | 0.7566 | 0.3463 | < 0.0001 | 0.996 |
| Age * Group | | | 0.9915 | 0.6752 | 0.9835 | 0.8021 | 0.0558 | 0.6625 | 0.96 | 0.9582 | 0.3202 | 0.0001 | < 0.0001 | < 0.0001 |
| Generation * Group *  Age | | | 0.681 | 0.8071 | 0.4619 | 0.3176 | 0.2714 | 0.8066 | 0.7823 | 0.9951 | 0.6845 | 0.3729 | < 0.0001 | 0.9828 |

The results are expressed as mean ± SEM. WGR – Specific weight gain rate; ∆AC – Abdominal circumference gain in cm; ∆NAL – Naso-anal length gain in cm; LI – Lee index; SI – Somatic index of each organ in %; VAT – Visceral adipose tissue; PAT – Perigonadal adipose tissue; FCTotal – Total food consumption in g; WCTotal – Total water consumption in mL; WGTIR – Weight Gain-to-Caloric Intake Ratio in g/kcal. p-Value obtained through three-way ANOVA followed by the post hoc multiple comparisons test (HSD-Tukey test).

**Table S2. Biometric analysis of male Wistar rats from the first (F1) and second (F2) generations exposed to residues of DDT.**

| Groups | | | **WGR** | **ΔAC** | **ΔNAL** | **LI** | **SI**  **Brain** | **SI**  **Liver** | **SI**  **Kidney** | **SI**  **VAT** | **SI**  **PAT** | **FC_Total_** | **WC_Total_** | **WGTIR** |
| --- | --- | --- | --- | --- | --- | --- | --- | --- | --- | --- | --- | --- | --- | --- |
| Generation F1 | PND35 | CTL | 2.02 ± 0.08 | 2.00 ± 0.54 | 5.55 ± 0.15 | 319.8 ± 2.0 | 1.41 ± 0.05 | 4.18 ± 0.12 | 0.51 ± 0.01 | 0.09 ± 0.02 | 0.24 ± 0.02 | 177 ± 13 | 420 ± 10 | 1,68 ± 0,14 |
|  |  | DDD | 1.87 ± 0.17 | 3.26 ± 0.21 | 4.85 ± 0.48 | 325.9 ± 6.6 | 1.39 ± 0.09 | 4.49 ± 0.29 | 0.54 ± 0.02 | 0.11 ± 0.02 | 0.26 ± 0.02 | 186 ± 5 | 333 ± 10 | 1,55 ± 0,19 |
|  |  | DDE | 2.21 ± 0.06 | 2.75 ± 0.24 | 4.81 ± 0.36 | 346.9 ± 12.2 | 1.31 ± 0.05 | 4.28 ± 0.12 | 0.48 ± 0.00 | 0.15 ± 0.01 | 0.26 ± 0.01 | 208 ± 2 | 360 ± 4 | 1,48 ± 0,03 |
|  |  | DDD/DDE | 1.96 ± 0.17 | 3.08 ± 0.39 | 5.20 ± 0.36 | 325.4 ± 7.2 | 1.32 ± 0.04 | 4.47 ± 0.17 | 0.49 ± 0.03 | 0.13 ± 0.02 | 0.24 ± 0.02 | 173 ± 4 | 440 ± 23 | 1,78 ± 0,09 |
|  | PND105 | CTL | 8.18 ± 0.27 | 7.57 ± 0.46 | 11.28 ± 0.36 | 332.8 ± 3.5 | 0.56 ± 0.02 | 3.35 ± 0.08 | 0.43 ± 0.01 | 1.23 ± 0.20 | 1.06 ± 0.05 | 1823 ± 33 | 3747 ± 67 | 0,61 ± 0,01 |
|  |  | DDD | 7.41 ± 0.40 | 7.63 ± 0.47 | 11.57 ± 0.32 | 325.6 ± 5.1 | 0.54 ± 0.03 | 3.48 ± 0.09 | 0.46 ± 0.01 | 1.16 ± 0.06 | 1.02 ± 0.10 | 1755 ± 15 | 3439 ± 19 | 0,63 ± 0,01 |
|  |  | DDE | 8.31 ± 0.26 | 7.92 ± 0.20 | 11.08 ± 0.33 | 338.1 ± 4.3 | 0.55 ± 0.01 | 3.45 ± 0.07 | 0.41 ± 0.00 | 1.34 ± 0.05 | 1.11 ± 0.07 | 1804 ± 20 | 2995 ± 19 | 0,64 ± 0,01 |
|  |  | DDD/DDE | 7.42 ± 0.49 | 8.13 ± 0.33 | 10.60 ± 0.55 | 336.1 ± 3.2 | 0.57 ± 0.02 | 3.36 ± 0.12 | 0.42 ± 0.00 | 1.42 ± 0.13 | 1.05 ± 0.04 | 1780 ± 84 | 3302 ± 42 | 0,62 ± 0,02 |
| Generation F2 | PND35 | CTL | 1.36 ± 0.09 | 1.75 ± 0.21 | 3.48 ± 0.25 | 345.4 ± 2.0 | 1.80 ± 0.04 | 4.28 ± 0.10 | 0.55 ± 0.02 | 0.12 ± 0.02 | 0.26 ± 0.01 | 134 ± 9 | 173 ± 6 | 1,54 ± 0,09 |
|  |  | DDD | 1.17 ± 0.24 | 1.49 ± 0.47 | 3.05 ± 0.54 | 338.4 ± 5.1 | 2.06 ± 0.21 | 4.10 ± 0.15 | 0.57 ± 0.03 | 0.10 ± 0.02 | 0.21 ± 0.04 | 134 ± 9 | 247 ± 21 | 1,29 ± 0,25 |
|  |  | DDE | 1.57 ± 0.06 | 2.42 ± 0.32 | 3.38 ± 0.56 | 358.1 ± 9.1 | 1.42 ± 0.02 | 4.35 ± 0.14 | 0.48 ± 0.01 | 0.13 ± 0.01 | 0.26 ± 0.01 | 134 ± 9 | 279 ± 8 | 1,79 ± 0,07 |
|  |  | DDD/DDE | 1.28 ± 0.12 | 2.27 ± 0.22 | 3.18 ± 0.09 | 356.2 ± 6.8 | 1.54 ± 0.05 | 4.30 ± 0.10 | 0.52 ± 0.02 | 0.14 ± 0.03 | 0.22 ± 0.04 | 134 ± 9 | 197 ± 7 | 2,03 ± 0,14 |
|  | PND105 | CTL | 7.83 ± 0.16 | 8.10 ± 0.37 | 11.71 ± 0.40 | 330.4 ± 2.3 | 0.54 ± 0.01 | 3.41 ± 0.06 | 0.41 ± 0.00 | 1.10 ± 0.02 | 1.04 ± 0.04 | 1749 ± 39 | 3057 ± 80 | 0,67 ± 0,01 |
|  |  | DDD | 7.58 ± 0.24 | 8.18 ± 0.44 | 12.08 ± 0.31 | 324.4 ± 4.5 | 0.52 ± 0.03 | 3.30 ± 0.04 | 0.42 ± 0.00 | 1.13 ± 0.13 | 1.13 ± 0.06 | 1786 ± 29 | 3066 ± 47 | 0,64 ± 0,02 |
|  |  | DDE | 7.46 ± 0.30 | 8.55 ± 0.17 | 12.13 ± 0.30 | 321.0 ± 2.8 | 0.56 ± 0.00 | 3.35 ± 0.06 | 0.41 ± 0.01 | 0.99 ± 0.08 | 1.09 ± 0.06 | 1755 ± 38 | 3579 ± 71 | 0,66 ± 0,01 |
|  |  | DDD/DDE | 7.10 ± 0.41 | 8.13 ± 0.44 | 11.71 ± 0.40 | 326.3 ± 5.8 | 0.51 ± 0.01 | 3.32 ± 0.06 | 0.43 ± 0.00 | 1.23 ± 0.12 | 1.03 ± 0.05 | 1891 ± 12 | 3124 ± 29 | 0,64 ± 0,01 |
| Generation | | | 0,0003 | 0,3352 | 0.0066 | 0.0336 | 0.0004 | 0.1928 | 0.4961 | 0.0587 | 0.9719 | 0.0703 | < 0.0001 | 0.5781 |
| Age | | | < 0,0001 | < 0,0001 | < 0.0001 | 0.0006 | < 0.0001 | < 0.0001 | < 0.0001 | < 0.0001 | < 0.0001 | < 0.0001 | < 0.0001 | < 0.0001 |
| Group | | | 0,0377 | 0,1407 | 0.7204 | 0.0181 | 0.0276 | 0.9158 | 0.0019 | 0.349 | 0.6304 | 0.6697 | 0.0244 | 0.045 |
| Generation * Age | | | 0,2155 | 0,0018 | < 0.0001 | < 0.0001 | < 0.0001 | 0.7798 | 0.1004 | 0.0593 | 0.61 | 0.0325 | 0.997 | 0.9632 |
| Generation * Group | | | 0,6511 | 0,3745 | 0.6587 | 0.2752 | 0.1379 | 0.1947 | 0.8356 | 0.6112 | 0.8862 | 0.1384 | < 0.0001 | 0.2481 |
| Age * Group | | | 0,3856 | 0,8007 | 0.2446 | 0.0602 | 0.0126 | 0.6888 | 0.2989 | 0.5721 | 0.9039 | 0.1081 | 0.007 | 0.036 |
| Generation * Group *  Age | | | 0,5588 | 0,393 | 0.8807 | 0.4263 | 0.131 | 0.6558 | 0.4765 | 0.6906 | 0.5263 | 0.0663 | < 0.0001 | 0.2138 |

The results are expressed as mean ± SEM. WGR – Specific weight gain rate; ∆AC – Abdominal circumference gain in cm; ∆NAL – Naso-anal length gain in cm; LI – Lee index; SI – Somatic index of each organ; VAT – Visceral adipose tissue; PAT – Perigonadal adipose tissue; FCTotal – Total food consumption in g; WCTotal – Total water consumption in mL; WGTIR – Weight Gain-to-Caloric Intake Ratio in g/kcal. p-Value obtained through three-way ANOVA followed by the post hoc multiple comparisons test (HSD-Tukey test).

**Table S3. Markers of tissue damage in female Wistar rats from the first (F1) and second (F2) generations exposed to residues of DDT.**

| Groups | | | **Cholinesterase** | | **GPT** | **Lipoperoxidation** | | | | **SOD** | | | **CAT** | | | **GPx** | | **GR** | | **GST** | |
| --- | --- | --- | --- | --- | --- | --- | --- | --- | --- | --- | --- | --- | --- | --- | --- | --- | --- | --- | --- | --- | --- |
|  |  |  | Brain | Liver | Liver | Liver | Kidney | VAT | PAT | Liver | Kidney | | Liver | | Kidney | Liver | Kidney | Liver | Kidney | Liver | Kidney |
| Generation F1 | PND35 | CTL | 6.82 ± 0.80 | 2.80 ± 0.15 | 4.31 ± 0.39 | 5.31 ± 0.76 | 2.91 ± 0.35 | 6.62 ± 1.03 | 1.85 ± 0.43 | 113.3 ±11.4 | | 33.6 ±3.7 | 88.2 ± 9.6 | 242.6 ±22.5 | | 242.0 ±26.4 | 296.0 ±25.5 | 3.99 ± 0.28 | 86.2 ± 5.4 | 111.5 ± 7.3 | 183.7 ±10.4 |
|  |  | DDD | 8.52 ± 1.55 | 3.42 ± 0.15 | 4.22 ± 0.27 | 4.49 ± 0.40 | 2.37 ± 0.35 | 6.49 ± 1.24 | 0.78 ± 0.12 | 96.5 ± 12.4 | | 30.1 ±2.6 | 57.0 ± 5.5 | 158.7 ±14.0 | | 233.1 ±27.7 | 240.4 ±23.5 | 3.99 ± 0.26 | 86.1 ± 9.4 | 120.4 ± 6.5 | 184.0 ±11.2 |
|  |  | DDE | 9.50 ± 2.59 | 3.28 ± 0.24 | 4.77 ± 0.67 | 5.61 ± 0.33 | 1.99 ± 0.32 | 4.04 ± 0.35 | 0.88 ± 0.03 | 109.5 ±14.2 | | 28.0 ±2.4 | 87.0 ±15.2 | 181.9 ±17.1 | | 245.5 ±22.7 | 225.1 ±23.7 | 4.25 ± 0.57 | 92.3 ± 4.6 | 127.4 ±10.1 | 182.6 ± 4.4 |
|  |  | DDD/DDE | 6.03 ± 0.53 | 2.82 ± 0.26 | 3.52 ± 0.18 | 4.01 ± 0.51 | 2.03 ± 0.24 | 6.76 ± 1.10 | 1.08 ± 0.07 | 110.9 ±11.1 | | 26.0 ±1.8 | 86.5 ±11.2 | 204.3 ±20.9 | | 287.2 ±34.9 | 269.3 ±25.5 | 4.40 ± 0.57 | 95.8 ± 6.3 | 122.1 ±11.2 | 181.4 ± 5.6 |
|  | PND105 | CTL | 8.63 ± 1.66 | 2.70 ± 0.25 | 3.96 ± 0.24 | 3.13 ± 0.41 | 3.25 ± 0.68 | 2.62 ± 0.29 | 1.53 ± 0.26 | 39.9 ± 1.4 | 13.4 ± 2.9 | | 48.9 ± 8.0 | 213.3 ±12.9 | | 395.1 ±66.8 | 439.6 ±40.5 | 3.17 ± 0.27 | 104.9 ±13.1 | 105.4 ± 5.0 | 126.3 ±13.9 |
|  |  | DDD | 8.11 ± 2.22 | 2.63 ± 0.03 | 3.99 ± 0.51 | 4.23 ± 0.17 | 2.69 ± 0.37 | 4.31 ± 0.83 | 1.68 ± 0.40 | 49.1 ± 3.7 | 9.9 ± 1.5 | | 48.1 ± 8.6 | 182.6 ±24.7 | | 395.8 ±71.3 | 569.2 ±28.0 | 4.01 ± 0.28 | 99.6 ± 12.0 | 120.2 ± 9.2 | 101.3 ± 4.9 |
|  |  | DDE | 10.20 ±1.82 | 2.48 ± 0.11 | 3.46 ± 0.51 | 4.50 ± 0.47 | 2.25 ± 0.41 | 3.02 ± 0.35 | 2.28 ± 0.22 | 46.4 ± 2.4 | 7.3 ± 1.4 | | 48.6 ± 7.1 | 200.5 ±20.3 | | 384.4 ±82.6 | 514.6 ±42.9 | 3.44 ± 0.33 | 114.3 ± 8.6 | 117.3 ±13.4 | 118.5 ± 5.3 |
|  |  | DDD/DDE | 9.59 ± 0.81 | 2.87 ± 0.22 | 3.59 ± 0.35 | 3.48 ± 0.26 | 2.52 ± 0.25 | 3.92 ± 0.88 | 1.51 ± 0.22 | 42.7 ± 1.5 | 13.7 ± 3.0 | | 61.9 ±12.5 | 202.6 ±13.9 | | 291.0 ±55.2 | 471.2 ±43.7 | 3.71 ± 0.38 | 108.5 ± 7.7 | 121.9 ± 7.5 | 117.2 ±11.3 |
| Generation F2 | PND35 | CTL | 9.89 ± 1.54 | 2.13 ± 0.14 | 3.13 ± 0.22 | 3.22 ± 0.54 | 2.53 ± 0.43 | 3.07 ± 0.37 | 1.48 ± 0.26 | 36.3 ± 2.0 | 13.9 ± 2.3 | | 60.2 ± 5.3 | 222.5 ±21.6 | | 306.4 ±21.2 | 383.6 ±49.1 | 3.45 ± 0.14 | 92.9 ± 9.7 | 109.6 ± 8.2 | 119.3 ± 6.0 |
|  |  | DDD | 11.13 ±1.32 | 2.22 ± 0.21 | 3.24 ± 0.21 | 3.24 ± 0.18 | 2.01 ± 0.46 | 3.79 ± 0.54 | 1.38 ± 0.22 | 40.2 ± 1.5 | 17.8 ± 2.3 | | 63.3 ±10.7 | 203.1 ±19.6 | | 284.5 ±20.5 | 436.7 ±19.4 | 3.98 ± 0.39 | 87.0 ± 6.4 | 89.9 ± 5.8 | 119.3 ± 4.2 |
|  |  | DDE | 9.65 ± 0.91 | 2.43 ± 0.17 | 3.59 ± 0.37 | 3.96 ± 0.55 | 1.72 ± 0.22 | 3.75 ± 0.54 | 2.01 ± 0.41 | 33.0 ± 2.0 | 19.1 ± 3.2 | | 79.8 ±10.7 | 163.8 ±15.2 | | 360.7 ±42.0 | 433.7 ±26.3 | 4.75 ± 0.43 | 69.5 ± 5.6 | 101.8 ± 7.8 | 118.3 ± 4.7 |
|  |  | DDD/DDE | 12.89 ±1.65 | 2.28 ± 0.20 | 2.63 ± 0.46 | 2.51 ± 0.42 | 2.97 ± 0.50 | 3.54 ± 0.92 | 1.74 ± 0.21 | 39.8 ± 3.3 | 14.2 ± 1.9 | | 53.7 ± 3.9 | 162.1 ±13.5 | | 307.8 ±27.8 | 338.3 ±24.4 | 3.68 ± 0.44 | 88.4 ± 4.2 | 98.7 ± 7.7 | 116.0 ± 3.5 |
|  | PND105 | CTL | 9.35 ± 0.75 | 2.51 ± 0.27 | 4.13 ± 0.43 | 4.27 ± 0.56 | 3.16 ± 0.33 | 2.61 ± 0.28 | 0.78 ± 0.03 | 59.8 ± 7.9 | 7.7 ± 1.8 | | 91.3 ±19.4 | 187.4 ±16.2 | | 446.8 ±67.3 | 368.3 ±41.6 | 5.77 ± 1.21 | 119.3 ± 4.8 | 129.2 ±13.6 | 102.7 ± 7.5 |
|  |  | DDD | 8.42 ± 1.59 | 2.75 ± 0.40 | 4.03 ± 0.31 | 3.81 ± 0.43 | 3.18 ± 0.39 | 3.60 ± 1.06 | 2.14 ± 0.70 | 69.6 ± 7.2 | 13.5 ± 2.1 | | 69.9 ± 9.3 | 174.0 ±18.1 | | 404.7 ±65.3 | 436.9 ±38.6 | 4.96 ± 1.31 | 118.0 ± 7.3 | 122.8 ±14.4 | 92.6 ± 10.1 |
|  |  | DDE | 7.53 ± 0.58 | 2.12 ± 0.22 | 3.62 ± 0.56 | 4.08 ± 0.31 | 3.14 ± 0.39 | 6.31 ± 1.14 | 3.36 ± 0.72 | 68.9 ± 6.1 | 19.3 ± 1.7 | | 72.7 ± 8.7 | 196.9 ±16.3 | | 381.2 ±34.3 | 573.6 ±36.0 | 4.43 ± 0.51 | 105.5 ± 6.5 | 81.6 ± 3.3 | 81.6 ± 7.0 |
|  |  | DDD/DDE | 9.02 ± 1.11 | 3.08 ± 0.11 | 6.74 ± 1.30 | 4.62 ± 0.31 | 3.69 ± 0.40 | 3.22 ± 0.47 | 0.96 ± 0.12 | 90.4 ± 2.7 | 15.4 ± 2.0 | | 98.3 ±16.1 | 196.5 ±21.3 | | 510.1 ±43.0 | 493.0 ±30.6 | 6.31 ± 1.11 | 118.2 ± 9.7 | 122.9 ±11.0 | 78.4 ± 2.5 |
| Generation | | | 0,0916 | < 0,0001 | 0.6638 | 0.0057 | 0.1271 | 0.0142 | 0.1062 | < 0.0001 | 0.0001 | | 0.2075 | | 0.2723 | 0.0069 | 0.0025 | 0.0274 | 0.7015 | 0.0253 | < 0.0001 |
| Age | | | 0,5574 | 0,6658 | 0.0813 | 0.7289 | 0.0006 | 0.0074 | 0.0322 | 0.0002 | < 0.0001 | | 0.3492 | | 0.9515 | < 0.0001 | < 0.0001 | 0.2401 | < 0.0001 | 0.3926 | < 0.0001 |
| Group | | | 0,9622 | 0,3387 | 0.8957 | 0.0644 | 0.0752 | 0.5848 | 0.0151 | 0.2513 | 0.8937 | | 0.1974 | | 0.0345 | 0.9390 | 0.0430 | 0.8482 | 0.5320 | 0.5023 | 0.2507 |
| Generation * Age | | | 0,0138 | 0,0012 | 0.0011 | < 0.0001 | 0.0876 | 0.0004 | 0.1787 | < 0.0001 | < 0.0001 | | 0.0002 | | 0.8908 | 0.9921 | < 0.0001 | 0.0078 | 0.0716 | 0.0674 | < 0.0001 |
| Generation *Group | | | 0,1962 | 0,4612 | 0.0831 | 0.4694 | 0.1085 | 0.0102 | 0.0209 | 0.3002 | 0.0013 | | 0.8112 | | 0.3866 | 0.6589 | 0.0592 | 0.9434 | 0.0785 | 0.0274 | 0.3264 |
| Age * Group | | | 0,7073 | 0,0311 | 0.0138 | 0.1307 | 0.9239 | 0.0763 | 0.0016 | 0.4209 | 0.1685 | | 0.3734 | | 0.1410 | 0.6500 | 0.0262 | 0.5001 | 0.8639 | 0.1103 | 0.5299 |
| Generation * Group * Age | | | 0,5634 | 0,4335 | 0.3137 | 0.2193 | 0.7970 | 0.8813 | 0.6942 | 0.4642 | 0.6516 | | 0.2382 | | 0.4292 | 0.1835 | 0.0802 | 0.3713 | 0.9708 | 0.4329 | 0.6851 |

The results are expressed as mean ± SEM. Cholinesterase expressed in nmol.min⁻¹.mg protein⁻¹; GPT (Glutamic Pyruvic Transaminase) expressed in nkat.mg protein⁻¹; Lipoperoxidation expressed in nmol MDA.mg protein⁻¹; SOD (Superoxide Dismutase) expressed in U.mg protein⁻¹; CAT (Catalase) expressed in µmol.min⁻¹.mg protein⁻¹; GPx (Glutathione Peroxidase), GR (Glutathione Reductase), and GST (Glutathione-S-Transferase) expressed in nmol.min⁻¹.mg protein⁻¹. p-value obtained through three-factor ANOVA followed by the post hoc multiple comparisons of means (HSD-Tukey Test).

**Table S4. Markers of tissue damage in male Wistar rats from the first (F1) and second (F2) generations exposed to residues of DDT.**

| Groups | | | **Cholinesterase** | | GPT | **Lipoperoxidation** | | | | **SOD** | | **CAT** | | **GPx** | | **GR** | | **GST** | |
| --- | --- | --- | --- | --- | --- | --- | --- | --- | --- | --- | --- | --- | --- | --- | --- | --- | --- | --- | --- |
|  |  |  | Brain | Liver | Liver | Liver | Kidney | VAT | PAT | Liver | Kidney | Liver | Kidney | Liver | Kidney | Liver | Kidney | Liver | Kidney |
| Generation F1 | PND35 | CTL | 12.38 ±1.91 | 2.75 ± 0.42 | 4.28 ± 0.53 | 5.39 ± 0.40 | 3.53 ± 0.83 | 13.4 ±1.18 | 0.21 ± 0.09 | 50.1 ± 3.4 | 29.0 ± 1.2 | 85.9 ± 11.7 | 297.9 ±39.6 | 267.5 ±29.3 | 246.8 ±19.1 | 6.04 ± 0.63 | 108.3 ± 3.7 | 96,7 ± 10,4 | 96,9 ± 3,4 |
|  |  | DDD | 15.15 ±1.60 | 2.88 ± 0.63 | 4.36 ± 0.40 | 5.21 ± 0.38 | 1.72 ± 0.34 | 9.20 ±1.09 | 2.99 ± 1.01 | 51.7 ± 4.0 | 29.1 ± 2.5 | 67.5 ± 17.0 | 400.8 ±27.6 | 224.0 ±44.0 | 261.2 ±19.0 | 5.60 ± 0.86 | 119.7 ±16.8 | 93,4 ± 11,6 | 106,8 ± 4,8 |
|  |  | DDE | 10.89 ±1.01 | 2.57 ± 0.26 | 5.59 ± 1.50 | 4.37 ± 0.26 | 1.54 ± 0.22 | 14.8 ±3.58 | 0.48 ± 0.14 | 47.2 ± 6.4 | 29.0 ± 2.5 | 86.8 ± 14.8 | 352.2 ±40.5 | 334.8 ±57.8 | 193.8 ±30.4 | 6.72 ± 0.89 | 128.2 ±11.7 | 100,1 ± 8,9 | 106,0 ±10,1 |
|  |  | DDD/DDE | 12.82 ±1.26 | 2.38 ± 0.19 | 4.07 ± 0.58 | 5.42 ± 0.46 | 2.17 ± 0.64 | 15.0 ±3.99 | 0.94 ± 0.48 | 52.5 ± 4.6 | 31.4 ± 1.3 | 79.0 ± 9.8 | 341.9 ±43.8 | 281.4 ±20.1 | 243.5 ±15.4 | 6.25 ± 0.51 | 116.0 ± 8.4 | 99,9 ± 10,8 | 96,2 ± 5,4 |
|  | PND105 | CTL | 8.15 ± 1.06 | 3.31 ± 0.36 | 9.86 ± 0.67 | 6.57 ± 0.35 | 6.13 ± 1.24 | 6.32 ± 2.45 | 0.45 ± 0.12 | 115.1 ± 9.2 | 21.0 ± 3.3 | 97.7 ± 13.7 | 391.7 ±70.3 | 324.9 ±38.7 | 334.7 ±23.5 | 7.00 ± 0.91 | 178.1 ±12.0 | 119,7 ± 8,1 | 139,4 ±13,0 |
|  |  | DDD | 7.87 ± 0.89 | 3.10 ± 0.52 | 7.49 ± 0.74 | 6.46 ± 0.64 | 6.28 ± 1.61 | 4.79 ± 1.21 | 2.63 ± 1.31 | 87.9 ± 21.1 | 21.8 ± 3.1 | 88.3 ± 8.4 | 366.2 ±51.8 | 331.0 ±22.5 | 307.5 ±44.1 | 5.63 ± 0.38 | 178.4 ±12.0 | 96,0 ± 9,0 | 131,3 ±18,7 |
|  |  | DDE | 8.49 ± 0.81 | 2.94 ± 0.39 | 8.61 ± 0.72 | 5.46 ± 0.36 | 3.81 ± 0.53 | 7.95 ± 1.66 | 1.75 ± 0.42 | 67.4 ± 6.7 | 25.5 ± 2.9 | 100.7 ± 8.0 | 482.7 ±23.2 | 381.9 ±26.0 | 351.5 ±17.3 | 6.65 ± 0.47 | 196.0 ±7.2 | 110,5 ± 4,8 | 119,6 ± 6,1 |
|  |  | DDD/DDE | 6.47 ± 0.73 | 4.02 ± 0.77 | 8.01 ± 1.55 | 5.49 ± 0.27 | 3.66 ± 0.60 | 6.13 ± 1.75 | 3.00 ± 0.66 | 84.6 ± 15.4 | 20.7 ± 3.1 | 73.3 ± 9.2 | 384.5 ±27.5 | 301.9 ±15.1 | 350.0 ±25.0 | 5.78 ± 0.59 | 198.3 ±14.1 | 106,2 ± 7,0 | 127,6 ±10,5 |
| Generation F2 | PND35 | CTL | 8.01 ± 1.21 | 2.40 ± 0.13 | 4.20 ± 0.45 | 6.49 ± 0.34 | 3.61 ± 0.58 | 3.23 ± 0.48 | 2.43 ± 0.87 | 100.5 ± 6.4 | 19.6 ± 0.7 | 73.1 ± 5.9 | 350.7 ±44.0 | 217.8 ±14.1 | 153.5 ±14.9 | 5.40 ± 0.28 | 124.9 ±18.2 | 84,3 ± 3,0 | 98,3 ± 9,5 |
|  |  | DDD | 9.12 ± 0.63 | 2.50 ± 0.16 | 4.74 ± 0.44 | 6.28 ± 0.35 | 2.95 ± 0.48 | 5.57 ± 1.25 | 4.31 ± 0.90 | 84.7 ± 5.5 | 26.2 ± 3.9 | 90.8 ± 6.7 | 363.2 ±27.7 | 234.7 ±30.2 | 157.3 ±19.7 | 6.03 ± 0.60 | 149.2 ±6.5 | 89,3 ± 7,1 | 91,7 ± 8,7 |
|  |  | DDE | 6.41 ± 0.33 | 2.98 ± 0.50 | 5.55 ± 0.40 | 5.97 ± 0.29 | 3.45 ± 0.67 | 7.27 ± 2.06 | 3.35 ± 1.18 | 88.3 ± 8.7 | 30.3 ± 1.7 | 109.0 ± 5.2 | 312.7 ±21.0 | 355.6 ±23.3 | 130.2 ± 7.9 | 7.74 ± 0.65 | 151.7 ±11.3 | 89,8 ± 5,6 | 75,9 ± 2,1 |
|  |  | DDD/DDE | 11.15 ±1.13 | 2.13 ± 0.06 | 6.18 ± 1.47 | 6.83 ± 0.37 | 2.62 ± 0.28 | 3.60 ± 0.81 | 1.10 ± 0.39 | 105.1 ± 9.4 | 19.6 ± 1.4 | 70.4 ± 9.3 | 288.3 ±31.7 | 239.3 ±13.4 | 134.1 ±13.9 | 5.54 ± 0.29 | 129.7 ±13.9 | 79,9 ± 3,4 | 96,7 ± 7,1 |
|  | PND105 | CTL | 6.28 ± 0.51 | 2.88 ± 0.25 | 6.90 ± 0.46 | 7.58 ± 0.45 | 3.81 ± 0.56 | 3.83 ± 0.90 | 2.53 ± 0.95 | 71.2 ± 6.4 | 21.9 ± 3.0 | 97.3 ± 4.9 | 355.5 ±25.2 | 404.7 ±12.6 | 275.9 ±23.6 | 5.97 ± 0.32 | 163.3 ±13.4 | 102,3 ± 4,5 | 117,3 ± 2,1 |
|  |  | DDD | 7.08 ± 0.63 | 3.17 ± 0.16 | 9.12 ± 0.91 | 7.03 ± 0.26 | 2.80 ± 0.49 | 5.07 ± 1.06 | 1.82 ± 0.45 | 69.9 ± 3.4 | 18.4 ± 2.2 | 101.9 ± 7.4 | 250.1 ±16.4 | 371.1 ±28.1 | 250.0 ±20.2 | 6.53 ± 0.34 | 150.2 ±10.9 | 113,5 ± 5,7 | 101,1 ± 5,4 |
|  |  | DDE | 8.73 ± 0.63 | 2.89 ± 0.21 | 8.62 ± 0.76 | 6.55 ± 0.44 | 3.32 ± 0.30 | 4.01 ± 0.49 | 2.15 ± 0.24 | 64.2 ± 1.9 | 16.8 ± 1.2 | 93.4 ± 8.1 | 229.3 ±32.1 | 408.8 ±27.5 | 243.1 ± 7.6 | 7.11 ± 0.85 | 133.5 ±5.5 | 108,9 ± 7,2 | 98,8 ± 3,1 |
|  |  | DDD/DDE | 7.74 ± 1.04 | 3.01 ± 0.19 | 7.36 ± 0.54 | 6.55 ± 0.22 | 4.63 ± 0.73 | 3.73 ± 0.37 | 1.73 ± 0.07 | 81.6 ± 6.5 | 19.0 ± 2.1 | 81.8 ± 12.0 | 273.6 ±25.5 | 417.0 ±10.3 | 232.3 ±20.0 | 6.55 ± 0.49 | 149.1 ±9.7 | 105,2 ± 4,7 | 95,8 ± 5,0 |
| Generation | | | < 0,0001 | 0,2003 | 0.8136 | < 0.0001 | 0.5438 | < 0.0001 | 0.0075 | 0.0004 | 0.0003 | 0.3303 | < 0.0001 | 0.1213 | < 0.0001 | 0.6736 | 0.1287 | 0.0879 | < 0.0001 |
| Age | | | < 0,0001 | 0,0008 | < 0.0001 | 0.0003 | < 0.0001 | < 0.0001 | 0.8943 | 0.1306 | < 0.0001 | 0.0732 | 0.8564 | < 0.0001 | < 0.0001 | 0.4287 | < 0.0001 | < 0.0001 | < 0.0001 |
| Group | | | 0,3388 | 0,9855 | 0.4943 | 0.0063 | 0.0597 | 0.2436 | 0.0161 | 0.0201 | 0.3547 | 0.0279 | 0.6977 | 0.0005 | 0.4895 | 0.0274 | 0.7680 | 0.7489 | 0.1282 |
| Generation * Age | | | 0,0004 | 0,6192 | 0.3885 | 0.3623 | 0.0012 | 0.0010 | 0.0057 | < 0.0001 | 0.3117 | 0.8326 | 0.0027 | 0.0039 | 0.7462 | 0.6589 | < 0.0001 | 0.0791 | 0.0526 |
| Generation *Group | | | 0,0720 | 0,4302 | 0.3260 | 0.7435 | 0.0655 | 0.1653 | 0.0575 | 0.2243 | 0.7227 | 0.3755 | 0.0297 | 0.9855 | 0.1951 | 0.2554 | 0.4292 | 0.2299 | 0.5266 |
| Age * Group | | | 0,0049 | 0,1579 | 0.6884 | 0.1182 | 0.6656 | 0.6492 | 0.0415 | 0.4855 | 0.3775 | 0.4825 | 0.0933 | 0.2608 | 0.5914 | 0.6354 | 0.1937 | 0.9497 | 0.4966 |
| Generation * Group * Age | | | 0,6581 | 0,5776 | 0.2776 | 0.9629 | 0.0607 | 0.6849 | 0.4359 | 0.1686 | 0.0086 | 0.3053 | 0.3900 | 0.2358 | 0.4126 | 0.6296 | 0.4318 | 0.6065 | 0.2652 |

The results are expressed as mean ± SEM. Cholinesterase is expressed in nmol.min⁻¹.mg protein⁻¹; GPT (Glutamic Pyruvic Transaminase) is expressed in nkat.mg protein⁻¹; Lipoperoxidation is expressed in nmol MDA.mg protein⁻¹; SOD (Superoxide Dismutase) is expressed in U.mg protein⁻¹; CAT (Catalase) is expressed in µmol.min⁻¹.mg protein⁻¹; GPx (Glutathione Peroxidase), GR (Glutathione Reductase), and GST (Glutathione-S-Transferase) are expressed in nmol.min⁻¹.mg protein⁻¹. p-value obtained through three-factor ANOVA followed by the Tukey HSD multiple comparisons test.
